# Inhibiting the endoplasmic reticulum stress response enhances the effect of doxorubicin by altering the lipid metabolism of liver cancer cells

**DOI:** 10.1016/j.molmet.2023.101846

**Published:** 2023-11-27

**Authors:** Maria Kopsida, Ada Lerma Clavero, Jaafar Khaled, David Balgoma, Clara Luna-Marco, Azazul Chowdhury, Sofi Sennefelt Nyman, Fredrik Rorsman, Charlotte Ebeling Barbier, Peter Bergsten, Hans Lennernäs, Mikael Hedeland, Femke Heindryckx

**Affiliations:** 1 Department of Medical Cell Biology, Uppsala University, Sweden; 2 Analytical Pharmaceutical Chemistry, Department of Medicinal Chemistry, Uppsala University, Sweden; 3 Unidad de Excelencia, Instituto de Biología y Genética Molecular (IBGM), Universidad de Valladolid – Consejo Superior de Investigaciones Científicas (CSIC), Valladolid, Spain; 4 Department of Surgical Sciences, Section of Radiology, Uppsala University, Uppsala, Sweden; 5 Department of Medical Sciences, Uppsala University, Uppsala, Sweden; 6 Translational Drug Development and Discovery, Department of Pharmaceutical Biosciences, Uppsala University, Sweden

**Keywords:** Lipidomics, Hepatocellular carcinoma, Endoplasmic reticulum stress, Chemotherapy

## Abstract

Hepatocellular carcinoma (HCC) is characterized by a low and variable response to chemotherapeutic treatments. One contributing factor to the overall pharmacodynamics is the activation of endoplasmic reticulum (ER) stress pathways. This is a cellular stress mechanism that becomes activated when the cell's need for protein synthesis surpasses the ER's capacity to maintain accurate protein folding, and has been implicated in creating drug-resistance in several solid tumors.

**Objective:**

To identify the role of ER-stress and lipid metabolism in mediating drug response in HCC.

**Methods:**

By using a chemically-induced mouse model for HCC, we administered the ER-stress inhibitor 4μ8C and/or doxorubicin (DOX) twice weekly for three weeks post-tumor initiation. Histological analyses were performed alongside comprehensive molecular biology and lipidomics assessments of isolated liver samples. In vitro models, including HCC cells, spheroids, and patient-derived liver organoids were subjected to 4μ8C and/or DOX, enabling us to assess their synergistic effects on cellular viability, lipid metabolism, and oxygen consumption rate.

**Results:**

We reveal a pivotal synergy between ER-stress modulation and drug response in HCC. The inhibition of ER-stress using 4μ8C not only enhances the cytotoxic effect of DOX, but also significantly reduces cellular lipid metabolism. This intricate interplay culminates in the deprivation of energy reserves essential for the sustenance of tumor cells.

**Conclusions:**

This study elucidates the interplay between lipid metabolism and ER-stress modulation in enhancing doxorubicin efficacy in HCC. This novel approach not only deepens our understanding of the disease, but also uncovers a promising avenue for therapeutic innovation. The long-term impact of our study could open the possibility of ER-stress inhibitors and/or lipase inhibitors as adjuvant treatments for HCC-patients.

## Highlights

• Through a pre-clinical model resembling human HCC, the study reveals a promising approach utilizing the ER-stress inhibitor 4μ8C to enhance the impact of DOX, resulting in improved drug response.
• The combination treatment of 4μ8C and DOX affects lipid turnover within tumor tissue, dampens oxygen consumption, and modifies the inflammatory microenvironment.
• The findings elucidate a crucial shift in cell fate mechanisms. DOX treatment induces ferroptosis, but when combined with 4μ8C, it promotes apoptosis.
• Combination treatment significantly lowers intracellular triglyceride levels and alters lipid metabolism, impacting the energy reserves of tumor cells.
• The study suggests that inhibiting IRE1 with 4μ8C could be an adjuvant therapy for HCC patients. Particularly during procedures like TACE-treatment, this approach could address the current limitations of chemotherapy.

## 1 Introduction

Hepatocellular carcinoma (HCC) is the most common primary liver malignancy and one of the leading causes of cancer-related mortality worldwide. Patient numbers are expected to rise due to the ongoing epidemic of obesity, which often leads to non-alcoholic fatty liver disease, one of the most common risk factors of HCC in the Western world. There is currently a shortage of effective chemotherapy for HCC [1,2]. Therefore, novel approaches to improve overall efficiency of existing treatments by increasing potency and/or reduce drug resistance are of uttermost importance.

The unfolded protein response (UPR) is an evolutionary conserved cell survival strategy and stress mechanism, initiated when the cell's need for protein synthesis exceeds the endoplasmic reticulum (ER)'s capacity to ensure precise protein folding [3]. In such cases, the accumulation of misfolded or unfolded proteins, known as ER-stress, is sensed through three ER-transmembrane proteins (IRE1α, PERK, ATF6), which activate the UPR with the goal of re-establishing normal ER-function [4,5]. It has been shown that key UPR-players are activated in the majority of HCC-patients and their expression has been correlated with poor prognosis [6,7]. In addition, activation of the UPR is considered to be responsible for the resistance to chemotherapy in solid tumors [8,9].

Numerous studies over the years have reported an aberrant accumulation of lipids in several types of cancer such as breast, lung and liver cancer, which were correlated with a more aggressive tumor phenotype [10,11]. Metabolic reprogramming is crucial for cancer cells to sustain tumor growth, as cancer cells need to produce sufficient ATP and intermediates for macromolecular biosynthesis, in order to meet requirements of intense and rapid cell proliferation [12]. While most healthy cells take up fatty acids from exogenous dietary sources; *de novo* fatty acid synthesis is the major source of cancer lipids, thereby providing a stable energy supply regardless of dietary fluctuations [10]. Interestingly, these metabolic adaptations can also contribute to a reduced response to chemotherapeutics [10]. Furthermore, there is a close link between activation of ER-stress pathways and alterations in lipid metabolism, as the ER serves as the main site of lipid production, and many enzymes involved in lipid metabolism are produced at that intracellular site [13]. The UPR is also known to directly regulate lipid homeostasis [13,14].

The aim of this study was to determine the impact of inhibiting IRE1α on lipid metabolism and how it influences the cytotoxic response to doxorubicin (DOX). This was achieved by using both *in vitro* and *in vivo* models, as we demonstrated that inhibition of IRE1α-endonuclease activity with 4μ8C can prevent chemoresistance by enhancing the cytotoxic effect of DOX by lowering the tumor cell's anabolic tone of biomolecules, including lipids, and depriving tumor cells from their energy reserves. The long-term impact of our study could open the possibility of ER-stress inhibitors as adjuvant treatments for HCC-patients.

## 2 Methods

### 2.1 Mouse model

A chemically-induced HCC mouse model was used [15,16]. Sixty male sv129-mice were allocated into five groups of 12 each. The first group served as healthy controls, receiving sham saline injections. The remaining four groups received intraperitoneal injections of DEN (35 mg/kg) every other week, for 28 weeks. Starting week 25, treatment groups received bi-weekly intraperitoneal injections of 4μ8C (10 μg/g), DOX (4 μg/g) intravenously, or a combination, for three weeks. At the study's end, mice were euthanized for sample collection. Our protocol adhered to the Uppsala ethical committee's standards for animal experimentation (DNR 5.8.18-0089/2020) and followed RESIST guidelines.

### 2.2 Cell culture

The three HCC cell lines (HepG2, ATCC® HB-8065™; SNU449 ATCC® CRL-2234™; Huh7, (kind gift from Dr. Mårten Fryknäs, Uppsala University, Sweden)) were cultured at 37 °C with 5% CO_2_ and 95% humidity within a CO_2_ incubator. The HepG2 and Huh7 were routinely cultured in Dulbecco's Modified Eagle Medium (DMEM) supplemented with 1% antibiotic antimycotic solution and 10% Fetal Bovine Serum (FBS). The SNU449 cell line was cultured in Roswell Park Memorial Institute (RPMI) 1640 medium supplemented with 1% antibiotic antimycotic solution and 10% FBS. Misidentification of all cell lines was checked at the Register of Misidentified Cell Lines, and none of the chosen cell lines were on the list. Extracted DNA from all cell lines were sent to Eurofins Genomics (Ebersberg, Germany) for cell line authentication using DNA and short tandem repeat-profiles. Authentication confirmed the correct identity of each cell line and each cell line tested negative for mycoplasma contamination. To determine the role of IRE1α and the effects of doxorubicin (DOX) *in vitro*, cells were treated for 24 h, cells with 10 μM 4μ8C (SML0949, Sigma Aldrich, Darmstadt, Germany) and/or 1 μM DOX (D558000, Toronto Research Chemicals, Canada). In the combination treatment, cells were pre-treated with 10 μM 4μ8C for 2 h and then changed to 1 μM DOX for 24 h. Cell proliferation was monitored *via* a resazurin reduction assay. Cells were seeded onto Corning® 96-well, flat, clear bottom, black plates (CLS3603-48EA, Sigma–Aldrich, Darmstadt, Germany) at a seeding density of 1.0 × 10^4^ cells per well. A 1% resazurin sodium salt solution (R7017-1G, Sigma–Aldrich, Darmstadt, Germany) was added in 1/80 dilution to the cells and incubated for 24 h, after which fluorescent signal was measured with a 540/35 excitation filter and a 590/20 emission filter on a Fluostar Omega plate reader.

### 2.3 Transfections

Nucleofection with 1 μM si-IRE1α (4390824, ThermoFisher Scientific, Stockholm, Sweden), or 1 μM siCtrl (4390843, ThermoFisher Scientific, Stockholm, Sweden) was performed using Amaxa Nucleofector program T-028 (HepG2) in ice-cold Ingenio electroporation solution (MIR50114, Mirus Bio LLC, Taastrup, Denmark) on 1.0 × 10^6^ cells per transfection. HepG2 cells were re-suspended in 2 mL DMEM with 10% FBS and left to adhere for 6 h, after which the medium was changed to fresh DMEM. Transfection efficiency was checked 24 h post-transfection by RT-PCR, achieving reduced mRNA expression by >60%.

### 2.4 Spheroid growth assays

Single cell solutions (2000 cells in 200 μL/well) were seeded in Nunclon Sphera-Treated, U-Shaped-Bottom, 96-well plates to generate spheroids (174925, ThermoFisher Scientific, Stockholm, Sweden). After the formation of spheroids (72 h), similar treatments to 2D experiments were applied. Cell viability was measured using the CellTiter-Glo® 3D kit (G9683, Promega), following manufacturer instructions.

### 2.5 Human liver cancer organoid isolation and culture

Needle biopsies were collected from tumors of six HCC patients prior to transarterial chemoembolisation with idarubicin on HCC patient (TACTida), a study conducted by Uppsala University. This clinical trial was approved by the Swedish Ethics Review Authority (Dnr. 2021-01928) as well as by the Medical Products Agency, Uppsala, Sweden (EUDRA: 2021-001257-31) and follows the principles of the Declaration of Helsinki. Informed consent was obtained from all patients. Needle biopsies were taken from tumors of HCC-patients at the discretion of the radiologist, and approximately 5 mg tissue sample was used for establishing organoid culture, as described by Nuciforo et al. [17]. Organoid culture was then initiated by mechanically and enzymatical dissociation of the tissue, following manufacturer's guidelines of the HepatiCult Organoid kit (Human) from Stemcell (Catalog #100-386, StemCell, Cambridge, UK). HepatiCult™ Organoid Initiation Medium (Catalog #100-0384, StemCell, Cambridge, UK) was used to efficiently generate hepatic organoids from different patients, which were further expanded and maintained in HepatiCult™ Organoid Growth Medium (Catalog #100-0385, StemCell, Cambridge, UK).

Tumor organoids splitting was performed at a density of 5 × 10^3^ cells in 15 mL Cultrex Reduced Growth Factor Basement Membrane Extract, Type 2, (BME2) droplets. Tumor organoids were treated with DOX, 4μ8C and Orlistat, similar as in the 2D cell culture experiments. Cell viability was measured after 1 day using CellTiter-Glo 3D reagent (Promega). Luminescence was measured on a Synergy H4 Multi-Mode Reader (BioTek Instruments).

### 2.6 Histological stainings

Tissue sections (8 μm) from paraffin-embedded blocks were stained with Hematoxylin and Eosin (H&E) and Sirius Red following standard procedures. Immunohistochemistry was performed with a horseradish peroxidase-DAB detection IHC-kit (ab64261, Abcam, Cambridge, UK) with antigen retrieval in a DIVA-decloaking chamber during overnight incubation. Protein Block was applied for 15 min, followed by a 2 h incubation at 37 °C with a Transferrin Receptor Polyclonal (Ref: PA5-27739, ThermoFisher Scientific, Stockholm, Sweden), ki67 (SolA15, ThermoFisher Scientific, Stockholm, Sweden) and Caspase-3 (ab184787, Abcam, Cambridge, UK). Images were acquired using a Nikon eclipse TE2000-U microscope equipped with a Nikon D-ECLIPSE camera and Nikon plan Apo objectives (Plan Fluor 10×/0,30 Ph1 DL). Pictures were obtained with Nikon ACT-1C for DXM1200C software and analyzed using Fiji/ImageJ. To detect the cell nuclei, thresholding was carried out on the blue section of color deconvoluted images, and the percentage area was then measured. Cell counts were assessed using the same color deconvolution setting. Thereafter, thresholding was performed as well as particle analysis at a pixel size of 5 per mm^2^. Quantifications of immunohistochemical stainings were conducted using color de-convolution to separate H-DAB staining.

For XBP1 (ab37152, Abcam, Cambridge, UK), IRE1α (ab37073, Abcam, Cambridge, UK), ATF4 (PA527576, ThermoFisher, Stockholm, Sweden) and PERK (PA582537, ThermoFisher, Stockholm, Sweden) overnight incubations were followed by a 60-minute incubation with secondary Donkey anti-rabbit Alexa Fluor-488 antibody (a21206, ThermoFisher, Stockholm, Sweden) and 8-minute Hoechst staining for cell nuclei. Images were captured using a Carl Zeiss LSM 700 Laser Scanning Microscope and analyzed with Fiji/Image J for fluorescence intensity.

### 2.7 Oil-red-O staining

Lipids were detected using a Lipid Oil Red O9 Staining Kit (MAK194, Sigma Aldrich, Darmstadt, Germany), following manufacturer's guidelines [18]. In brief, HepG2-cells were fixed in 10% formalin for 60 min. After 5 min incubation with 60% isopropanol, cells were covered with Oil Red O-Working Solution for 20 min. Counterstaining was performed with hematoxylin for 1 min. Pictures were taken with Nikon INC microscope (40× magnification). Lipid droplets were quantified with Fiji/ImageJ and image analysis was based on color de-convolution and threshold adjustment, allowing detection of lipid particles.

### 2.8 ER-tracker staining

HepG2 cells were stained with ER-Tracker™ Red (BODIPY™ TR Glibenclamide; E34250, ThermoFisher, Stockholm, Sweden), Calcein-AM (LIVE/DEAD™ Viability/Cytotoxicity Kit; L 3224 ThermoFisher, Stockholm, Sweden) and NucBlue™ Live ReadyProbes™ Reagent (Hoechst 33342) (R37605, ThermoFisher, Stockholm, Sweden). Images were taken with a confocal laser scanning microscope (Carl Zeiss LSM 700 Laser Scanning Microscope, Jena, Germany) using a Plan-Apochromat 10×/0,45 (Zeiss) objective. Fluorescence intensity was quantified with Fiji/Image J.

### 2.9 Quantitative RT-PCR of mRNA

RNA was isolated from animal tissue or cell culture using the E.Z.N.A. Total RNA-Kit I (R6834-02, Omega Bio-tek, Inc., Norcross, Georgia, USA) following manufacturer's guidelines. Quality and quantity of RNA was evaluated using Nanodrop. The iScript cDNA-synthesis kit (1708891, Bio-rad, Solna, Sweden) was used according to the manufacturer's protocol. Primers were designed using Primer Blast and ordered from ThermoFisher (Supplementary Material 1). Fast SYBR Green (Ref: 4385612, ThermoFisher Scientific) was used according to manufacturer's guidelines. Quantification of gene expression was done using QuantStudio 5 (ThermoFisher Scientific). Normalization of mRNA-expression was performed to *GAPDH* and/or *18S.* Average CT-values of two technical duplicates for each sample were calculated to determine fold change using the delta-delta-CT approach.

### 2.10 SDS-PAGE and western blot

Protein lysates in lysis buffer were mixed with 2× Laemmli buffer and heated to 95 °C for 5 min before being loaded onto a Precast Mini-Protean® TGXTM gels (456-9034, Biorad, Solna, Sweden). After separation, proteins were transferred to an Immobilon®-Fl membrane (IPFL0010, Millipore, Solna, Sweden). The membrane was blocked using the Intercept® (TBS) blocking buffer (927-60001, Li-Cor, Bad Homburg, Germany) diluted 1:4 in PBS, and then incubated with primary and secondary antibodies. After primary and secondary antibody incubation the membrane was washed 3 × 15 min in PBS-T (PBS + 0.1% Tween®20). Primary antibodies used were ATF4 (PA5-27576, Invitrogen, Stockholm, Sweden), XBP1 (ab37152, Abcam, Cambridge, UK) or Vinculin (14-9777-82, ThermoFisher, Stockholm, Sweden), diluted in blocking buffer with 0.1% Tween®20. Secondary antibody used was goat anti-rabbit IgG HRP (31460, ThermoFisher, Stockholm, Sweden) diluted in blocking buffer with 0.1% Tween®20 and 0.01% SDS. All incubations were carried out at room temperature for 1 h or overnight at 4 °C. The membranes were scanned using an Odyssey scanner (LI-COR Biotechnology).

### 2.11 Lipidomics

After randomization, approximately 10 mg of tissue was homogenized with a FastPrep-24 5G homogenizer (Lysing Matrix D, MP Biomedicals) at 4.0 m/s for 30 s. A volume equivalent to 1 mg of tissue was extracted by Bligh & Dyer-method. The chloroformic phases were mixed, evaporated under vacuum and resuspended in 200 μL of acetonitrile/isopropanol 50:50. Lipidomics was carried out as described before [19]. Samples were injected on an Acquity UPLC coupled to a Synapt G2 Q-ToF (Waters) with electrospray ionization. Injection order was randomized and a quality control built with a mixture of the resuspensions was injected every seven samples. The samples were analyzed in both positive and negative modes. Lipids were identified as described before [19,20], with a limit of absolute deviance of the *m/z* of 10 ppm. Subsequently, for every sample, the signal of a lipid was normalized to the total signal of lipids of in that sample. To eliminate potential effect of feeding on the lipid patterns, the fold change was calculated between the amount detected in the tumor tissue and the surrounding healthy (stromal) tissue for each sample. Preprocessed data can be found in Supplementary Material 3.

### 2.12 Oxygen consumption measurements

The basal oxygen consumption rate (OCR) was measured in HepG2-cells (25000 cells/well) cultured on Seahorse plates (101085-004, Agilent) for 24 h at 37 °C and 5% CO_2._ Mitochondrial respiration was determined by measuring the OCR in the Extracellular Flux Analyzer XF96e (Seahorse Biosciences). Assays were performed in XF-assay medium (Seahorse Biosciences) at pH 7.4 and supplemented with 25 mM glucose. Basal OCR was measured during 30 min and then, 4μ8C, DOX and 4μ8C + DOX were added from the injection port. OCR was measured over time for 2 h and then inhibitors of electron transport chain, rotenone (2 μM) and antimycin (2 μM), were added. This allowed quantification of non-mitochondrial OCR, which was used for normalization.

### 2.13 Reactive oxygen species (ROS) assay

ROS-generation was measured using DCFDA-Cellular ROS-Detection Assay Kit (ab113851, Abcam, Cambridge, UK), following manufacturer's guidelines. Fluorescence was measured at 485 nm excitation and 535 nm emission wavelengths, using a Fluostar Omega plate reader. Results of the microplate assay are shown as fold change fluorescence from six biological replicates.

### 2.14 Triglyceride assay

Five mg of healthy or tumorous tissue was weighted and processed following the instructions of the Triglyceride Kit (ab178780, Abcam, Cambridge, UK). Fluorescence intensity was measured in a Synergy H4 hybrid reader (Ex/Em = 535/587 nm, BioTek Instruments, USA). Results from the TG-assay were normalized to protein content determined with a Pierce™ BCA Protein Assay Kit (UE284352, ThermoFisher Scientific, Stockholm, Sweden), following manufacturer's instructions.

### 2.15 Human Protein Atlas

Patient survival was correlated to the expression levels of the selected perilipins and lipases by using publicly available data from the Human Protein Atlas [21]. Patients were classified into two expression groups based on the fragments per kilobase of exon per million reads (FPKM) value of each gene. The expressions of UPR-associated markers in the different tumor stages of HCC were taken from the Human Protein Atlas database using the RNA sequence data from 365 patients derived from the Cancer Genome Project. The dataset derived from the Human Protein Atlas was imported into GraphPad Prism 8. Based on the data from the Human Protein Atlas, the FPKM value of the appropriate gene was used as a cut-off to determine high or low expression. The FPKM/cut-off value corresponded to the best expression cut-off level, meaning that the FPKM value that would yield the maximum difference in regard to survival between the two groups at the lowest log-rank *p*-value. Statistical significance between the groups of the Kaplan–Meier curves was determined with a log-rank test. *p*-values of <0.05 were considered statistically significant. Representative images of healthy livers and HCC biopsies stained with antibodies against perilipins were derived from the Human Protein Atlas, in order to visualize protein expression.

### 2.16 Statistical analysis

Data are presented as mean ± standard deviation. Statistical significance was determined using one-way analysis of variance (ANOVA) followed by Tukey's multiple comparison test. *p*-values <0.05 were considered statistically significant. Statistical analyses and graphs were made using GraphPad Prism 9.

## 3 Results

### 3.1 Combination of DOX with inhibition of the IRE1α-endoribonuclease activity reduces tumor burden and alters the lipidome in a mouse model for HCC

**Figure 1:**
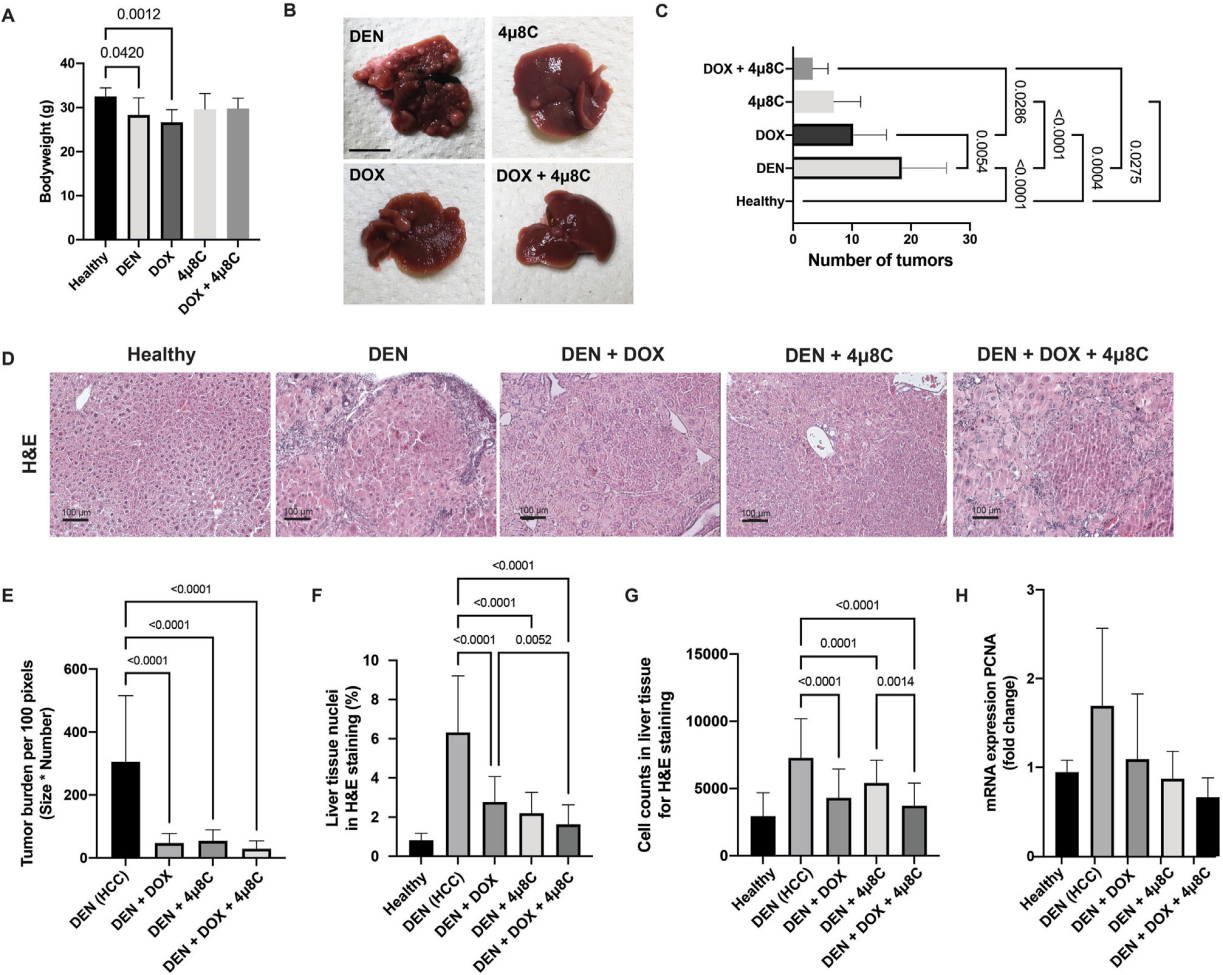
**Treatment with DOX and 4μ8C affects body weights and number of tumors in a DEN-induced mouse *in vivo* model for HCC**. (A) Bodyweight of mice. (B) Representative pictures of mice livers. (C) Number of macroscopic tumors. (D) Representative H&E-stained liver slides. (E) Tumor burden measured on H&E-stained slides. (F) Percentage of nuclei in liver tissue. (G) Number of cells in H&E-stained liver tissue. (H) mRNA expression of proliferation marker PCNA. N = 12 mice per group (A–G) or 5 per group (H). Scale bars represent 1 cm (B) or 100 μm (D).

Mice with DEN-induced HCC demonstrated a notable decline in body weight compared to their healthy counterparts (Figure 1A), similar to previous studies [16,22]. Administration of DOX exacerbated this weight reduction, aligning with DOX's recognized side effect of inducing weight loss [23] (Figure 1A). Intriguingly, treatment involving 4μ8C restored body weight levels of mice with DEN-induced HCC, to those observed in healthy mice. This suggests an enhancement in the overall health and integrated physiology of the mice (Figure 1A). These outcomes were mirrored by macroscopic analysis of the mice's livers, which unveiled a significant reduction in tumor count across all treatment groups (Figure 1B and C). Notably, co-administration of 4μ8C further amplified the anti-tumoral impact of DOX (Figure 1B and C) in mice with DEN-induced HCC.

**Figure 2:**
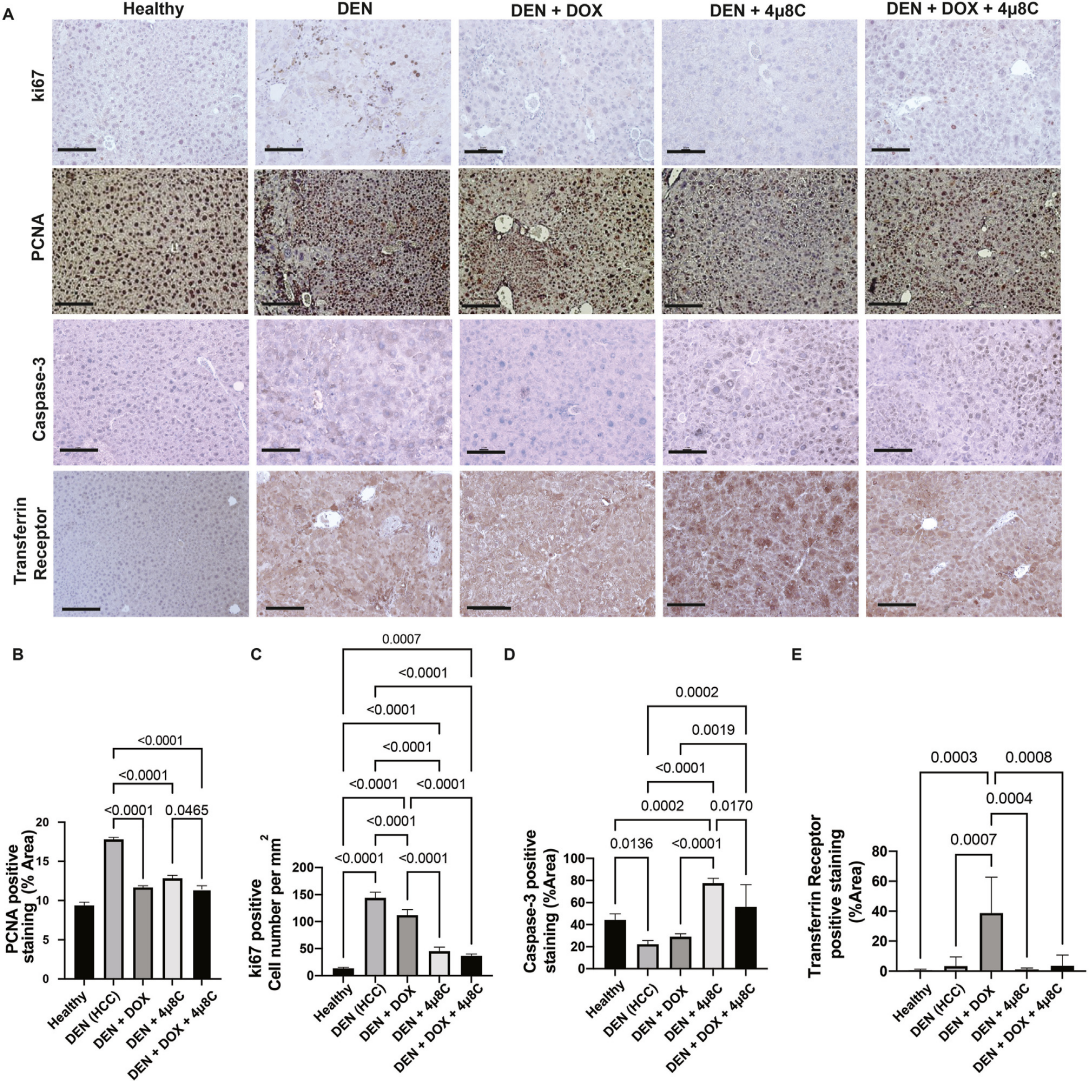
**Treatment with DOX and 4μ8C reduces proliferation and increases cell death.** (A) Representative images of immunohistochemical stainings using antibodies targeting ki67, PCNA, caspase 3 and transferrin receptor. (B) Quantification of the PCNA-positive area. (C) Quantification of the number of ki67 positive cells per mm^2^. (D) Quantification of caspase-3 positive staining area on liver slides. (E) Quantification of the transferrin receptor positive staining area on liver slides. N = 12 mice per group, scale bars represent 100 μm.

Histological analyses of liver samples from mice with DEN-induced HCC confirmed a decreased tumor burden following treatment with 4μ8C, DOX or the combination of 4μ8C + DOX, compared to untreated mice with DEN-induced HCC (Figure 1D and E). As tumors are characterized by highly proliferative areas, automated counting of nuclei can be used as a marker for HCC [24], thus the percentage and number of nuclei were counted on H&E-stained liver slides (Figure 1F and G). This analysis showed that DEN-induced HCC mice had an elevated number of cell nuclei, indicative of tumor presence. Notably, all treatments, especially the combined 4μ8C + DOX therapy, significantly decreased the number of nuclei, suggesting a reduction in tumor cell proliferation and overall tumor burden (Figure 1F and G). mRNA-expression of proliferation marker PCNA was also increased in DEN-induced livers compared to healthy tissue (Figure 1H). Combination of 4μ8C + DOX slightly reduced this marker in mice with DEN-induced HCC, although not significantly (Figure 1H). The effects of the different treatments were further assessed using immunohistochemical stainings for ki67, PCNA, Caspase-3 and Transferrin receptor (Figure 2A). As expected, DEN-induced HCC notably increased the area of PCNA-staining (Figure 2B) and the number of ki67 positive cells (Figure 2C), which was significantly reduced by all treatments, particularly by the 4μ8C + DOX combination. Caspase-3 staining revealed a decrease in apoptosis due to DEN, whereas 4μ8C and 4μ8C + DOX treatments markedly increased apoptosis compared to controls (Figure 2D). No increase of apoptosis was seen in the DOX-mono-treated livers with DEN-induced HCC, which could suggest an alternative form of cell death. As DOX is known to induce ferroptosis [20], we quantified transferrin receptor positive cells in liver tissue using immunohistochemistry. This showed that DOX increased transferrin receptor-positive staining, suggesting a possible increase in ferroptosis. Interestingly, treatment with 4μ8C reduced this effect (Figure 2E).

### 3.2 Treatment with DOX and 4μ8C reduces fibrosis and alters the inflammatory niche of the tumor microenvironment

**Figure 3:**
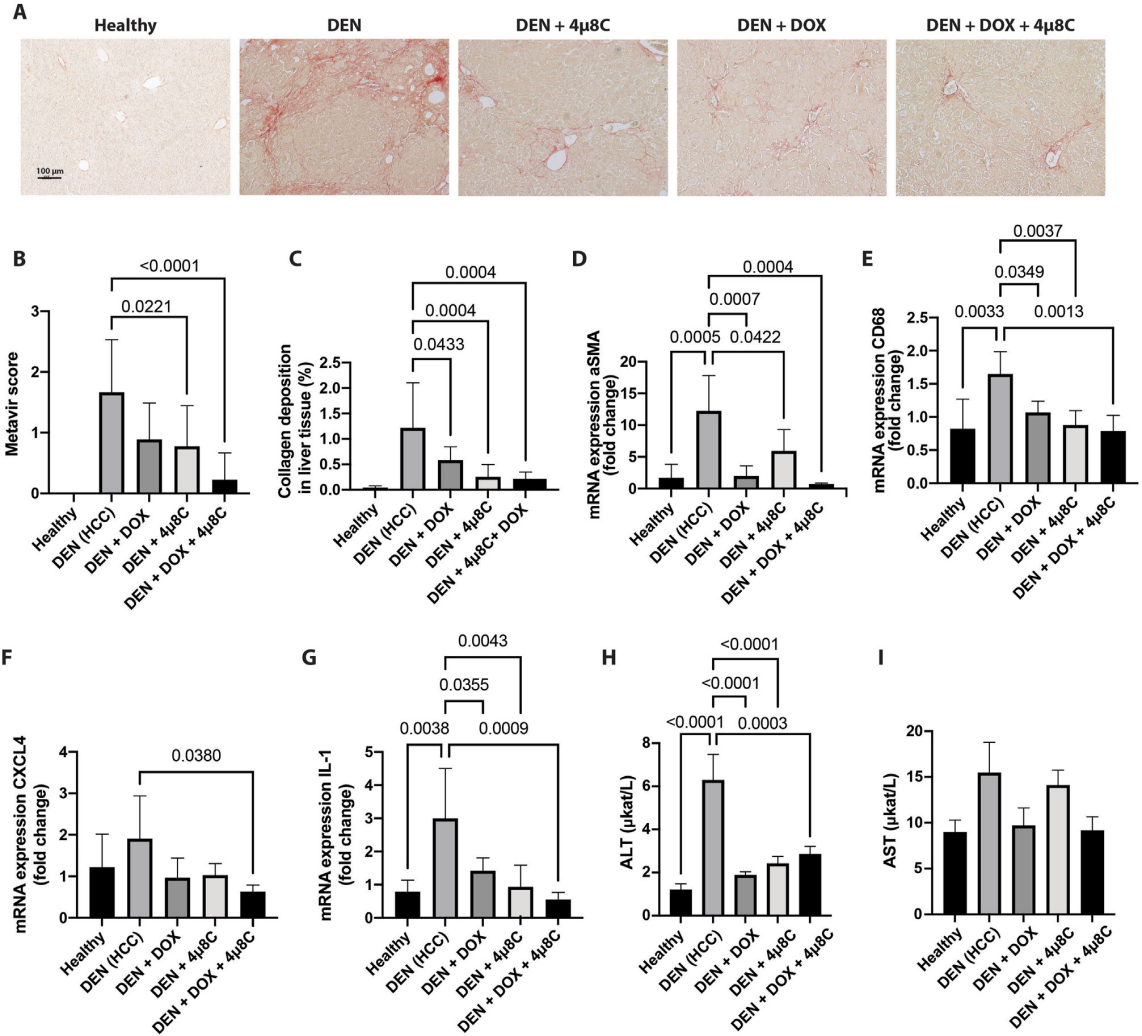
**Treatment with DOX and 4μ8C reduces fibrosis and inflammation**. (A) Representative images of sirius red stained liver slides. (B) Quantification of Metavir score and (C) Percentage of collagen deposition measured on Sirius red stained liver slides. mRNA-expression of (D) aSMA (E) CD68, (F) CXCL4 and (G) IL-1. Serum levels of (H) AST and (I) ALT. N = 12 mice per group, for qPCR N = 5 mice per group.

Our previous studies have shown that 4μ8C reduces fibrosis [25,26], which is known as a limiting factor in HCCs response to DOX [27,28]. Collagen accumulation, evident in Sirius red staining (Figure 3A) and higher Metavir scores (Figure 3B) in HCC mice, was diminished following 4μ8C, DOX, or their combination. This was also confirmed by quantifying collagen deposition on the same slides (Figure 3C). Similarly, mRNA-levels of the activated stellate cell marker, smooth muscle actin (aSMA), were elevated in HCC tumors, but were significantly decreased after treatments, especially with the combination therapy (Figure 3D). Further analyses of the mRNA-expression of inflammatory markers in the hepatic non-tumoral stroma showed a significant increase of overall inflammation in mice with HCC, which was reduced after the different treatments (Figure 3E–G). There was a significant increase in the monocyte-macrophage marker CD68 in stromal tissue of mice with HCC, compared to healthy controls (Figure 3E). This was significantly reduced after treatment with 4μ8C, DOX or the combination (Figure 3E), thus suggesting a decreased infiltration of macrophages. CXCL4 is known to be secreted by a variety of immune cells and has been implicated in several inflammatory and fibrotic diseases. We found that combinational treatment with both DOX + 4μ8C reduced CXCL4-expression in mice with a DEN-induced HCC (Figure 3F). Furthermore, IL-1 mRNA levels were notably lower in treated groups compared to untreated DEN-induced HCC (Figure 3G). Evaluating liver function, we noted higher alanine aminotransferase (ALT) and Aspartate aminotransferase (AST) serum levels in mice with DEN-induced HCC, indicative of liver damage (Figure 3H and I). The different treatments effectively lowered ALT levels towards levels observed in healthy mice (Figure 3H).

**Figure 4:**
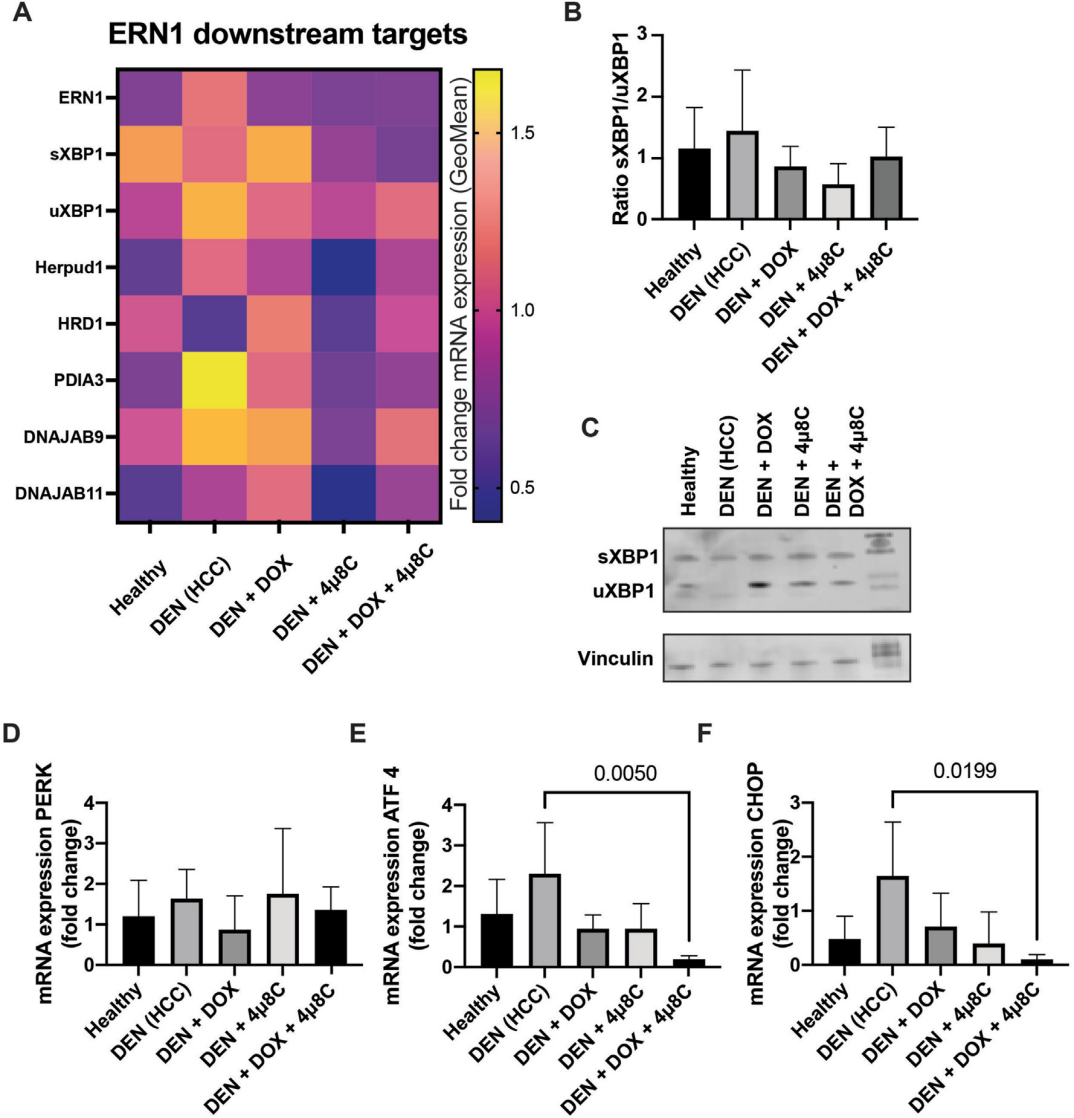
**Combination treatment with DOX and 4μ8C reduced expression of various ER-stress markers in liver tissue.** (A) mRNA levels of ERN1 and its downstream targets (sXBP1, uXBP1, Herpud1, HRD1, PDIA3, DNAJB9, DNAJB11). (B) Ratio of spliced and unspliced XBP1 mRNA (C) western blot of spliced and unspliced XBP1, with vinculin as loading control. mRNA levels of (D) PERK, (E) ATF4 and (F) CHOP. N = 5 mice per group.

To assess how these treatments affected ER-stress pathways and to confirm whether 4μ8C indeed suppressed the UPR, we analyzed the mRNA expression of ERN1 and associated downstream targets (sXBP1, uXBP1, Herpud1, HRD1, PDIA3, DNAJB9, DNAJB11). The heatmap shows changes in gene expression patterns induced by DEN combined with DOX, 4μ8C, or both, compared to DEN alone and healthy mice (Figure 4A). Notably, the DEN + 4μ8C treatment resulted in the most pronounced reduction in both ERN1 and its downstream targets, indicating a potential suppression of the UPR in this group. The ratio of mRNA expression of spliced to unspliced XBP1 remained consistent across groups (Figure 4B), implying that mRNA transcription ratios of sXBP1 and uXBP1 alone may not fully represent UPR activity, possibly due to the unconventional splicing mechanisms that have been observed [29]. Western blot analysis revealed that only the spliced isoform was present in liver lysates of mice with DEN-induced HCC, while the unspliced isoform was not detected in these samples (Figure 4C). This active splicing suggests UPR activation *via* the IRE1α pathway (Figure 4C). In contrast, treatment groups showed both spliced and unspliced forms, indicating a potential restoration of normal UPR activity. While PERK-mRNA levels remained constant in all treatment conditions, we found that combining DOX with 4μ8C reduced ATF4 and CHOP-levels compared to untreated HCC levels (Figure 4E and F). This observation aligns with prior studies indicating that DOX suppresses ER-stress activation [30,31]. Fluorescent immunohistochemistry additionally validated the elevated protein expression levels of IRE1α, XBP1, and ATF4 in DEN-induced tumors (Supplementary material 2), which restored after treatment with 4μ8C.

**Figure 5:**
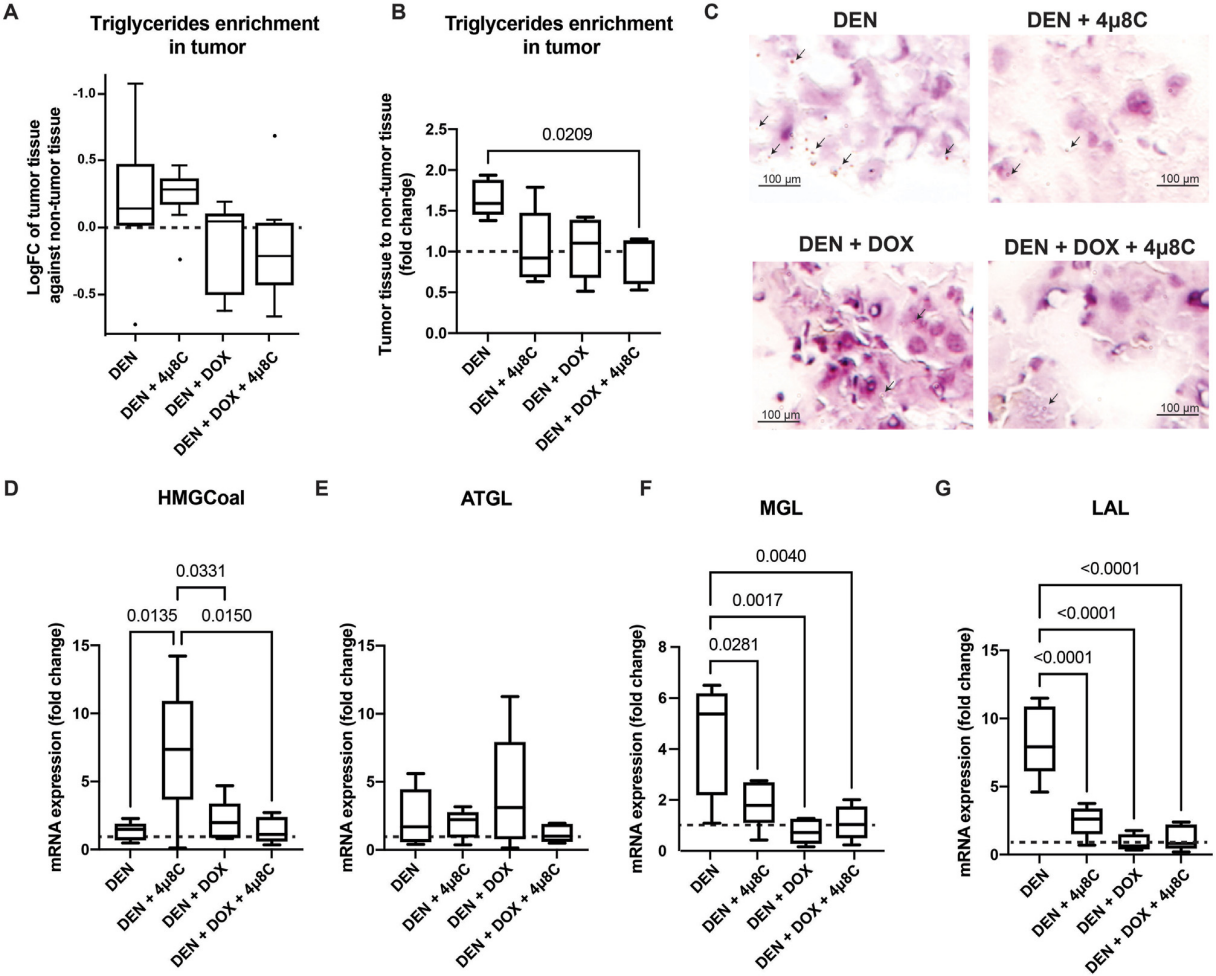
**Treatment with DOX and 4μ8C reduces triglyceride content of liver tumors and alters lipid metabolism.** (A) Triglyceride content of tumors measured through liquid chromatography-high resolution mass spectrometry and (B) *via* a commercial fluorimetric triglyceride assay. (C) Representative pictures of Oil-Red-O staining. mRNA expression of (D) HMG-CoA-reductase (E) ATGL, (F) MGL and (LAL). N = 5 mice per group. Fold change is compared to matched non-tumoral tissue, marked as dashed line in graphs.

Both DOX and ER-stress are known to affect the lipid composition of tumor tissue from mice, which can affect tumorigenesis, proliferation, resistance and drug response. Therefore, we mapped out the lipidome of tumor tissues of all treatment groups using liquid chromatography–high resolution mass spectrometry (Supplementary Material 3 and Figure 5A) and confirmed hepatic lipid contents through a commercial fluorometric triglyceride (TG) assay kit (Figure 5B), as well as Oil-Red-O staining (Figure 5C). Results were normalized to total signal of lipids in the surrounding non-tumoral tissue of the same animal, as to avoid effects caused by feeding patterns. These analyses revealed an overall enrichment of TGs in the tumor tissue (Figure 5A and B). However, treatment with DOX or 4μ8C seems to shift this pattern towards an overall reduction in TGs within the tumor tissue, which is even further decreased in the combination treatment with DOX + 4μ8C (Figure 5A and B). This was confirmed by Oil-Red-O staining, revealing an accumulation of lipid droplets in untreated tumor tissue, and a subsequent reduction when mice were treated with DOX + 4μ8C (Figure 5C). To unravel the mechanism by which DOX + 4μ8C shifts the triacylglycerides between tumor and surrounding non-tumoral tissue, the mRNA-expression of key enzymes involved in lipophagy (lysosomal acid lipase, LAL), *de novo* lipogenesis (fatty acid synthase, FAS; and glycerol-3-phosphate acyltransferase-1, GPAT-1), lipolysis (adipose triglyceride lipase, ATGL; and monoacylglycerol lipase, MGL) and fatty acid oxidation (PPARα) were measured (Table 1 and Figure 5D–G). In addition, as cholesterol constitutes lipid droplets together with triglycerides (Figure 5C), the mRNA-expression of HMG-CoA reductase (HMG-CoAR) was also measured.

**Table 1:** Summary of lipidomic changes in the tumor tissue after different treatments.

Gene	Function	Treatments							
		DEN		DEN + 4μ8C		DEN + DOX		DEN + DOX + 4μ8C	
HMG-CoAR	Lipid synthesis	1.3	↑	7.3	↑↑↑↑	2.1	↑↑	1.4	↑
FAS	Lipid synthesis	2.3	↑↑	2.9	↑↑	3.5	↑↑	1.4	↑
GPAT	Lipid synthesis	4.7	↑↑↑	1.0	≈	2.4	↑↑	1.8	↑
PPAR	FA oxidation	2.2	↑↑	2.4	↑↑	2.7	↑↑	2.5	↑↑
ATGL	Lipolysis	2.4	↑↑	1.9	↑	4.1	↑↑↑	1.2	↑
MGL	Lypolysis	4.4	↑↑↑	1.9	↑	0.75	↓	1.1	≈
LAL	Lipophagy	8.4	↑↑↑↑	2.5	↑↑	0.88	↓	1.2	≈
TOTAL ENRICHTMENT TGs			↑		↑↑		↓		↓↓

Overall, these data suggest that DOX + 4μ8C-combination treatment reduced the difference of mRNA-expression of genes involved in lipid metabolism between tumors and non-tumoral tissue (Table 1), thus normalizing the tumor's metabolism towards a non-tumoral state. While the average fold changes of mRNA-expression in tumors from the control group was higher than 2 (except for HMG-CoAR, Table 1, highlighted in Figure 5D), the average fold changes of mRNA-expression in tumors from the DOX + 4μ8C group were between 1 and 2 (except for PPARα) (Table 1). This effect was less clear in the mono treatments with DOX or 4μ8C, respectively. Compared to untreated tumors, this suggests a deceleration of lipid metabolism in tumors in the combination DOX + 4μ8C-group, thereby restoring values close to healthy levels. This deceleration did not affect the lipidomic snapshot (Supplementary Material 3), suggesting that DOX + 4μ8C decreased the anabolic and catabolic lipid pathways in tumors in a way that they cancel out in the lipidome. Despite the similar static lipidome, a deceleration of lipid-trafficking towards closer values to healthy tissue suggest more similar metabolism between tumors and healthy tissue [32]. This slowing metabolism and its potential impact on tumor bioenergetics may partially explain the decrease in tumor number by the combination treatment. Specifically, DOX + 4μ8C induced a normalization of ATGL, MGL, and LAL mRNA-expression towards normal levels (Table 1, graphically highlighted in Figure 5E–G). As the three enzymes are involved in the liberation of FAs from TGs, this reduction suggests a lower availability of FAs for β-oxidation. β-Oxidation of fatty acids plus the oxidation of acetyl-CoA by the citric acid cycle are intensive in the production of FADH2, NADH, and ATP. Consequently, similar levels ATGL, MGL, and LAL in tumors and healthy tissue of animals treated with DOX + 4μ8C suggest a lower level of reducing agents and a lower anabolic tone for all biomolecules, not only for lipids. This lower anabolic tone for all biomolecules may partially elucidate the decrease in the number of tumors in animals treated with DOX + 4μ8C.

**Figure 6:**
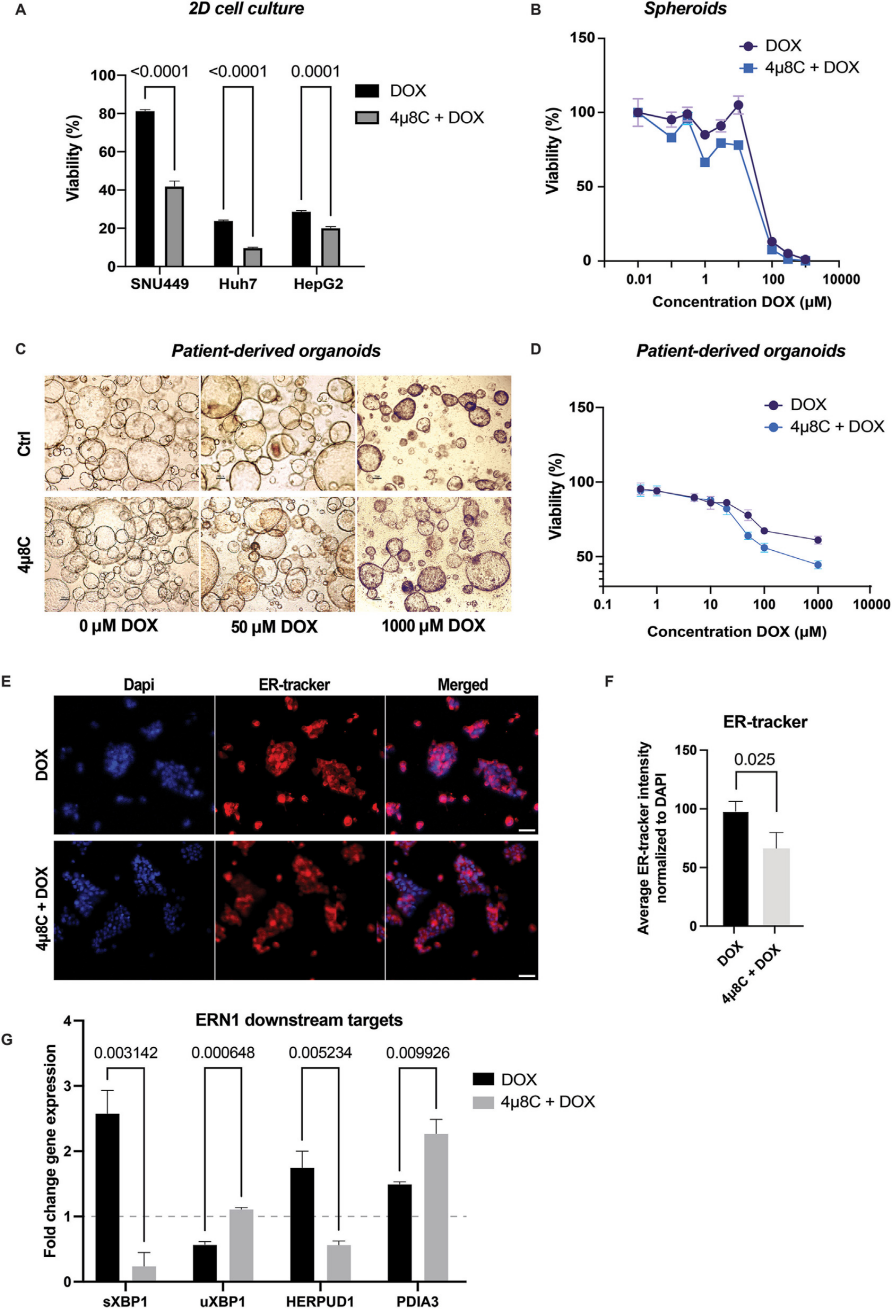
**Treatment with 4μ8C improves cytotoxic response to doxorubicin *in vitro***. (A) Cell viability of HCC-cell lines treated with DOX or the combination of DOX and 4μ8C. HepG2-cells were then chosen for further experimentation. (B) Cell viability of HepG2-spheroids treated with DOX or the combination of DOX and 4μ8C. (C) Representative images of patient-derived organoids treated with DOX or the combination of DOX and 4μ8C. (D) Cell viability of patient-derived organoids treated with DOX or the combination of DOX and 4μ8C. (E) Representative images of HepG2-cells stained with ER-stracker and Dapi. (F) Quantification of ER-tracker staining normalized to DAPI. (G) mRNA expression of downstream targets of ERN1, sXBP1, uXBP1, HERPUD1 and PDIA3. N = 3–8 biological replicates.

We then investigated the synergistic effect of DOX and 4μ8C on cell viability in different *in vitro* experiments, using HCC cell lines, spheroids, and patient-derived organoids. Specifically, SNU449, Huh7 and HepG2 cell lines were exposed to DOX alone or in combination with 10 μM 4μ8C for 24 h (Figure 6A). The combination with 4μ8C potentiated the cytotoxic effect of DOX in all three HCC-cell lines (Figure 6A). The HepG2 cell-line was used for further investigation and cell viability was assessed on HepG2-spheroids (Figure 6B). The results demonstrated that the combination of 4μ8C and DOX enhanced the cytotoxicity of DOX against HepG2-spheroids (Figure 6B). Subsequently, in our experiments with patient-derived primary organoids, exposure to varying concentrations of DOX naturally resulted in cellular toxicity, as visible on representative images (Figure 6C). Similarly, the combination treatment augmented DOX cytotoxicity in these patient-derived organoids (Figure 6D).

To delve into the underlying mechanism of this synergistic effect, we used HepG2-cells as a model system. HepG2-cells were stained with a fluorescent ER-tracker, and the ER size was normalized to the nucleus, in order to quantify ER expansion, a well-known downstream effect of XBP1 activation [33] (Figure 6E). The analysis revealed a significant reduction in ER size after 4μ8C treatment, indicating the inhibition of the IRE1α-XBP1 pathway (Figure 6F). Additionally, mRNA-expression of ERN1 downstream targets showed a decrease in sXBP1 and an increase in uXBP1 in HepG2-cells treated with 4μ8C, which implies reduced splicing activity (Figure 6E). HERPUD1 levels decreased, while PDIA3 increased with 4μ8C treatment (Figure 6E), further supporting the successful modulation of the ER-stress response with 4μ8C.

**Figure 7:**
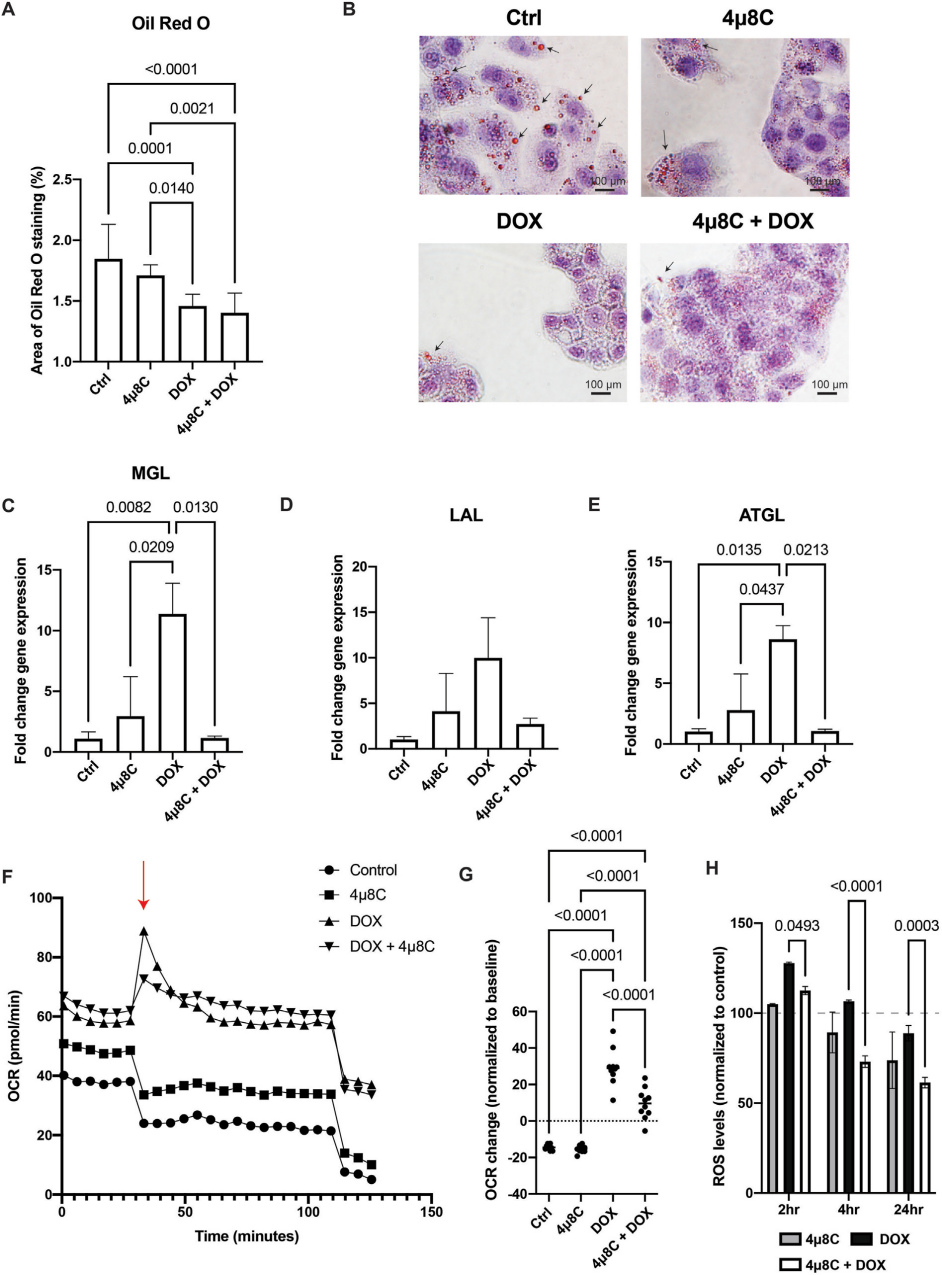
**Treatment with DOX and 4μ8C alters lipid metabolism of liver cancer cells *in vitro***. (A) Quantification of Oil-Red-O staining. (B) Representative images of Oil-Red-O staining. mRNA-expression of (C) MGL, (D) LAL and (E) ATGL. (F) Oxygen consumption rate of cells treated with DOX, 4μ8C or combination. Red arrow represents the time of drug exposure, while blue arrow represents the time of rotenone/antimycin injection to completely inhibit the OCR. (G) Oxygen consumption rate normalized to baseline levels. (H) ROS-levels normalized to untreated controls. N = 3–8 biological replicates.

Based on the *in vivo* data, we hypothesized alterations in the lipid metabolism lowered the lipid energy reserves of tumor cells and that this could have contributed to the synergistic effect of DOX with 4μ8C. Oil-Red-O staining confirmed that all treatments reduced intracellular lipid droplets in HepG2-cells, with the strongest reduction in the combination group (Figure 7A and B). The combination treatment also decreased mRNA-expression levels of MGL, LAL and ATGL (Figure 7C–E) compared to DOX-treatment alone (although not significantly in case of LAL, Figure 7D). This could suggest a lower level of reducing agents and a lower anabolic tone for all biomolecules. This lower anabolic tone could reduce the metabolic rate of cells, and could thereby contribute to limiting their growth. When cells were treated with DOX, we noted an increase of MGL (Figure 7C) and ATGL (Figure 7E) expression, as well as an increase in the oxygen consumption rate (OCR) (Figure 7F and G). All these parameters were significantly reduced when DOX was given in combination with 4μ8C. As metabolic activity and OCR leads to generation of reactive oxygen species (ROS), we measured time-dependent ROS-generation after exposure to DOX, 4μ8C or the combination (Figure 7H). As expected, DOX-exposure led to a rapid increase of ROS after 2 h, which gradually decreased towards base-line levels at later time-points. However, combination with 4μ8C significantly reduced ROS-generation compared to DOX alone in all time-points (Figure 7H), which taken together with the data from the OCR suggests that the combination treatment could have led to a lower bioenergetic state of the cells. It also suggests that the ROS pathway to the overall cytotoxic effect has been overemphasized and/or another cytotoxic mechanism(s) has/have become more important.

To ascertain the specificity of 4μ8C's effects on IRE1α, we used siRNA to knockdown ERN1 in HepG2 cells. ERN1-mRNA levels were reduced by over 70% post-siRNA transfection (see Supplementary Material 4A). This knockdown was mirrored by a corresponding decrease in mRNA-levels of ERN1-downstream targets, including sXBP1 (Supplementary Figure 4B). Additionally, ERN1-silencing led to a decrease in ER-size, as evidenced by ER-tracker staining (Supplementary Figure 4C–D). Overall, this suggests we successfully silenced the ERN1-XBP1-pathway.

Transfection with si-ERN1 significantly increased DOX-sensitivity, as the IC50 concentration of DOX in mock-transfected HepG2 cells was 41.24 μM (± 8.406), while si-ERN1 drastically reduced the IC50 value to 2.524 μM (± 0.562) (Supplementary material 4E–F). Oil-Red-O staining indicated that treatment with DOX reduced lipid droplets in both transfected and mock-transfected HepG2-cells, with the strongest reduction in the transfected group (Supplementary material 4G–H). Treatment with DOX also led to decreased levels of MGL and ATGL in transfected HepG2-cells (Supplementary material 4I), suggesting a lower level of reducing agents and a lower anabolic tone for biomolecules.

### 3.3 Lipid accumulation reduces response to doxorubicin

**Figure 8:**
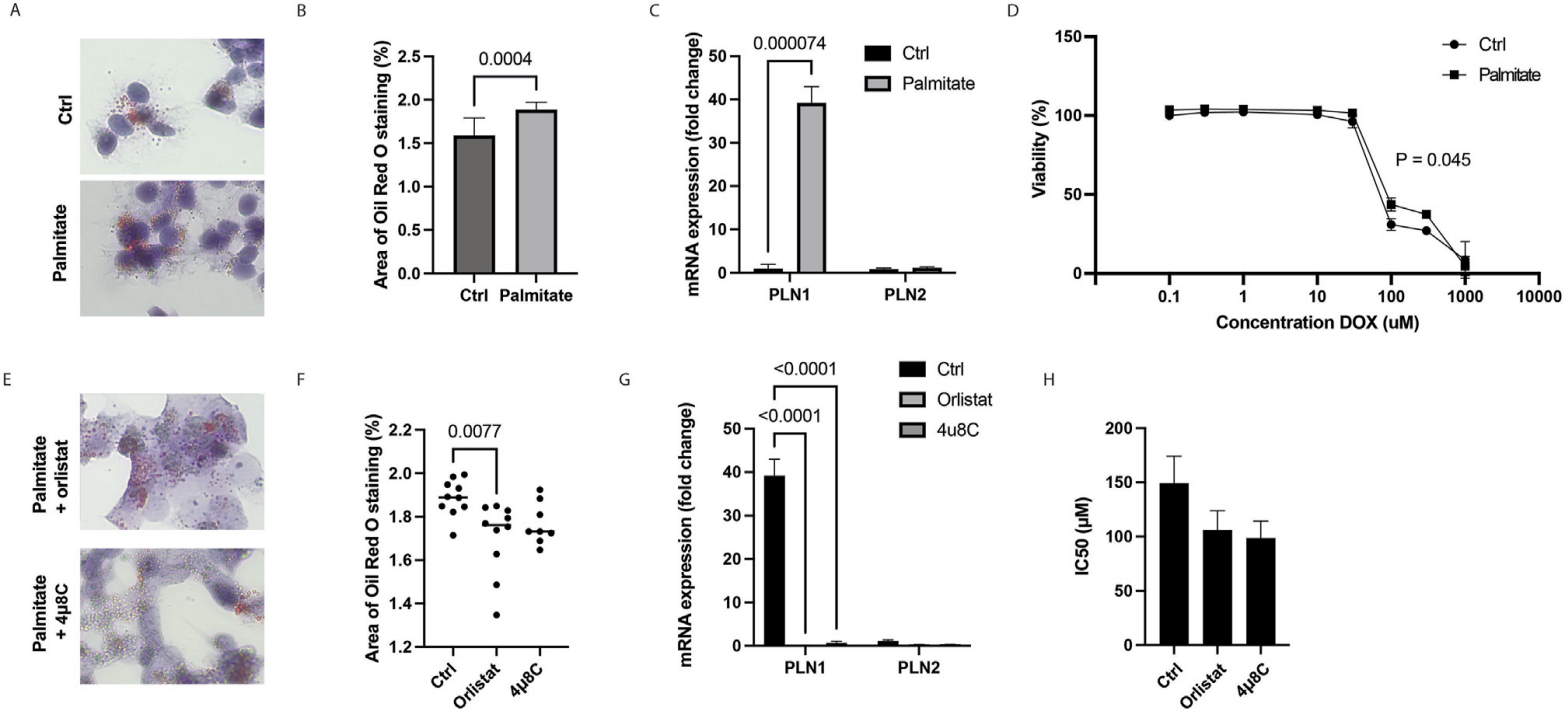
**Lipid accumulation reduces response to doxorubicin, which is reversed by treatment with orlistat and 4μ8C.** (A) Representative images of Oil-Red-O staining after treatment with palmitate. (B) Quantification of Oil-Red-O staining. (C) mRNA-expression of PLN1 and PLN2. (D) Cell viability of HepG2-cells after treatment palmitate and various concentrations of DOX. (E) Representative images of Oil-Red-O staining after treatment with orlistat and 4μ8C. (F) Quantification of Oil-Red-O staining. (G) mRNA-expression of PLN1 and PLN2 after treatment with orlistat and 4μ8C. (H) IC50 values after treatment with orlistat and 4μ8C in HepG2-cells.

In order to assess the impact of cellular bioenergetic state on the response to DOX, HepG2-cells were treated with palmitate, which increased the accumulation of intracellular lipid droplets (Figure 8A and B). Furthermore, palmitate treatment resulted in a significant increase of PLN1 mRNA-expression by nearly a 30-fold, while not affecting PLN2-expression (Figure 8C). In line with our hypothesis, palmitate treatment significantly decreased sensitivity to DOX, as the IC50-concentration of DOX in HepG2 cells was 94.64 μM (± 17.563), while palmitate increased the IC50-value to 149.3 μM (± 24.780) (Figure 8D). This suggests that the intracellular accumulation of lipid droplets may increase the energy reserve of tumor cells, as more lipids would be available for hydrolysis, thereby fueling tumor growth and possibly contributing to the recovery of DOX-treated cells. We therefore used a general lipase inhibitor, orlistat, to block lipase activity in palmitate-treated HepG2-cells and assessed the effect on lipid droplet accumulation and response to DOX. Orlistat significantly reduced lipid droplet accumulation in the HepG2-cells treated with palmitate, and a similar – yet non-significant – effect was seen by 4μ8C (Figure 8E–F). Both orlistat and 4μ8C inhibited the mRNA-expression of PLN1 in palmitate-treated HepG2 cells, but had no effect on PLN2 expression (Figure 8G). Interestingly, orlistat reduced the IC50 value to 106.12 μM (± 17.71), with a similar reduction in the 4μ8C-treated HepG2 cells (98.69 μM ± 15.722) (Figure 8H).

### 3.4 Expression levels of lipid accumulation markers and lipases correlate with poor prognosis in patients with HCC

In order to investigate the clinical relevance of our findings, we examined the expression of lipid accumulation markers in HCC-patients using the Human Protein Atlas. Specifically, we focused on the expression of perilipins, an evolutionary conserved family of lipid droplet-associated proteins, which are embedded in a phospho-lipid monolayer of intracellular lipid droplets. All three perilipins, PLIN1, PLIN2 and PLIN3, were increased in HCC-tissues compared to healthy tissues (Supplementary material 5A). Using The Cancer Genome Atlas RNA-expression data, we identified that while high expression of PLN1 was associated with a positive prognosis (Supplementary material 5B), increased expression of PLN2 (Supplementary material 5C) and PLN3 (Supplementary material 5D) was significantly associated with lower 5-year survival rate. In order to put our findings on lipases and orlistat in a clinical context, we also looked at survival of patients with a low or high expression of ATGL, MGL and LAL (Supplementary material 5E–G). Here we found that increased expression of these lipases is associated with poor survival, albeit not significant in the case of ATGL. These results suggest that increased lipid accumulation and increased hydrolyses of fats may contribute to a more aggressive and resistant tumor phenotype in HCC.

## 4 Discussion

Hepatocellular carcinoma (HCC) is the most common primary liver cancer and one of the leading causes of cancer-related mortality worldwide. There is currently a shortage of effective chemotherapy for HCC, due to lack of tumor target specificity, severe side-effects caused by drug toxicity and HCC's intrinsic resistance to chemotherapeutic agents [1]. Therefore, novel medical approaches to improve drug-response are of uttermost importance. By using an *in vivo* pre-clinical model known for its similarity to human HCC, we show that using the ER-stress inhibitor 4μ8C can potentiate the cytotoxic effect of DOX, by affecting the lipid turnover of tumoral tissue, lowering the oxygen consumption rate and by altering the inflammatory niche of the surrounding micro-environment. These novel results are promising and might be possible to translate into clinical trials in HCC patients in the near future.

The tight link between response to DOX and ER-stress pathways has been previously shown. For instance, studies have found that DOX downregulates ATF4-mRNA-expression [31], which explains the reduction of CHOP after treatment with both 4μ8C and DOX. Furthermore, inhibition of the PERK-ATF4-pathway can increase ferroptosis in cancer cells [34] and we have previously shown that DOX induces ferroptosis in HCC-cell lines and activates ER-stress-pathways [20]. Now, we quantified transferrin receptor in liver tissue and showed that DOX indeed increased the area of transferrin receptor positive staining, suggesting a possible increase in ferroptosis. This is further confirmed by the DOX-induced generation of ROS, which can sustain lipid peroxidation by regenerating Fe^2+^ from Fe^3+^ [35]. Interestingly, 4μ8C-treatment reduced both ROS-levels and transferrin receptor staining, which is in line with previous findings that ferroptosis is – at least in part – dependent on ER-stress pathways [36]. However, the combination of 4μ8C + DOX significantly increased apoptosis compared to untreated mice, as quantified through caspase-3 staining. These data suggests that DOX alone seems to mainly induce ferroptosis, while the combination of DOX + 4μ8C pushes the cells more towards apoptosis. In addition, adding 4μ8C also decreased the proliferation rate of the surviving cells, spheroids and patient-derived organoids. Therefore, the anti-tumoral effect of the combination treatment can be explained firstly, by the shift from ferroptosis to apoptosis and secondly, by reducing cell proliferation of non-responding tumor cells that did not undergo cell death. Possibly, this reduction in cell proliferation could be attributed by the decreased intracellular triglyceride levels and alterations in lipid metabolism, which reduced the cell's energy reserves.

Since ER-stress pathways and DOX have been widely described to be involved in regulating lipid metabolism in the liver and other organs [12,20,37,38] we investigated the effect of DOX and 4μ8C on the lipidome and on mRNA-expression of genes involved in lipid synthesis, oxidation, lipolysis, and lipophagy. In untreated DEN-induced animals, tumors presented strong increases in the expression of genes associated to anabolism (lipid synthesis) and catabolism (lipolysis, lipophagy, and lipid oxidation). While this might seem contradictory, this is typical for the aberrant tumoral metabolism [39]. Despite these changes, lipidomic profile did not show strong alterations (Supplementary Material 3), except for the intracellular enrichment in TGs. The similar lipidomic composition between tumors and surrounding non-tumor tissue, together with the general increase in mRNA-expression of lipid-related enzymes suggest that, from a general point of view, anabolism and catabolism cancel out in the static “photo” of lipidomics of DEN-induced tumors. However, the increase in anabolic and catabolic lipid enzymes suggest a much more intense trafficking of lipids in tumor tissue, when compared with normal tissue, which could provide an energy reserve for tumor cells that are known to have high metabolic needs. For TGs, the activity increases of the enzymes responsible for their synthesis (FAS and GPAT in our panel) may overcome the activity increase of the enzymes responsible for their degradation (ATGL and LAL in our panel). The net balance resulted in an intra- and extracellular enrichment in TGs in tumors in the animals carrying a liver cancer after being induced with DEN.

Treatment with 4μ8C did not notably affect the differences between tumor and healthy tissue, except for HMG-CoAR, MGL, and LAL. First, LAL is responsible for lipophagy, which partially catabolizes lipid droplets [39]. Consequently, the decrease in the tumor fold change of LAL in 4μ8C-treated animals may partially explain the stronger enrichment in TGs of the tumors from 4μ8C-treated animals. In addition, it is known that the IRE1α deletion provokes hepatic TG accumulation under the induction of ER-stress by tunicamycin [40]. This accumulation has been explained by impaired VLDL-secretion [37]. Therefore, the stronger accumulation of TGs in tumors from 4μ8C-treated animals might also be explained by a stronger impairment of VLDL-secretion in tumor cells than in healthy tissue. Second, MGL increase in tumors from 4μ8C-treated animals was lower than in control animals. MGL promotes cell proliferation and tumor growth [41], thus this might have contributed to the reduced proliferation rate and reduced tumor burden in our mouse model. Third, in contrast to LAL and MGL, HMG-CoAR was strongly more enhanced in tumors of animals treated with 4μ8C. The induction of cholesterol by IRE1α siRNA has been reported before [14]. In this context, our results suggest that this effect on HMG is stronger in tumor than in healthy tissue.

Treatment with DOX did not sensibly change the fold changes of the control tumors (DEN group), except for MGL and LAL. While control tumors (DEN group) showed average fold changes 4.4 and 8.4 times for MGL and LAL, the treatment with DOX presented much lower differences with average fold changes 0.75 and 0.88, respectively. Unexpectedly, the opposite was seen *in vitro*, yet it is important to note that the *in vitro* values were not normalized to healthy (baseline) levels, as the control experiments here were done with HCC-cell lines. As expressed before, the decrease of MGL after 4μ8C-treatment, might partially explain the decrease in number of tumors induced by DOX. To the best of our knowledge, it is the first time that someone shows that this affects the levels of LAL. As LAL not only hydrolyzes intracellular TGs in lipoproteins, but also TGs from extracellular lipid droplets [41]. In our study, we show that DOX-mono treatment simultaneously decreased tumoral expression, while increasing expression of LAL-mRNA-expression in surrounding non-tumoral tissue. This could suggest an increase in lipophagy in the surrounding tumor microenvironment. Further mechanistic studies are warranted to understand the potential role of lipophagy in the cytostatic effect of DOX in clinical practice.

In individuals with fatty liver disease, notably non-alcoholic fatty liver disease (NAFLD) and non-alcoholic steatohepatitis (NASH), significant changes in lipid profiles occur, characterized by the abnormal accumulation of lipids, including TGs, within liver cells. These alterations in lipidomic profiles align with the findings of our study. Dysregulated lipid metabolism, involving increased lipogenesis (lipid synthesis) and modified lipolysis (lipid breakdown), contributes to the increased risk of HCC in individuals with fatty liver disease. Consequently, our research, highlighting the role of ER-stress in modulating lipid metabolism, holds promise for identifying novel therapeutic targets aimed at preventing and treating HCC in patients with fatty liver disease. Further investigation, particularly in more specific models involving high-fat diets, is strongly warranted to explore the potential benefits of combining ER-stress response inhibitors with chemotherapy. Such targeted approaches may offer innovative avenues for intervention, ultimately leading to improved outcomes in this rapidly expanding patient population.

We also observed a significant alteration in the inflammatory environment after treatment with 4μ8C, DOX or the combination. CD68^+^ hepatic macrophages are associated with a strong phagocytic activity in response to inflammatory injury [42]. In our study, we observe a significant increase of CD68^+^ in stromal tissue of mice with HCC, which is in line with previous findings in HCC-patients [43]. Treatment with 4μ8C, DOX or the combination reduced the infiltration of CD68^+^-macrophages in the peritumoral tissue, to levels that were similar of those in healthy controls. Interestingly, studies have shown that CD68^+^ is implicated in the crosstalk between macrophages and fibroblasts in HCC [44]. As we have shown in previous studies and in this study, 4μ8C can decrease stellate cell activation, which could have affected endosialin expression in peritumoral tissues and therefore lead to a decreased recruitment of CD68^+^ macrophages. In addition, CXCL4 has also been proposed as a key factor driving innate immunity and forming the pivotal link between inflammation and fibrosis [45]. As we only observed a significant reduction of CXCL4 mRNA-expression in mice receiving both DOX + 4μ8C, this further supports the important anti-inflammatory and anti-fibrotic role of 4μ8C in HCC.

To summarize, we show that inhibiting IRE1α-endonuclease activity with 4μ8C can enhance the cytotoxic effect of DOX by lowering the tumor cell's anabolic tone of biomolecules, including lipids, and thereby deprive tumor cells from their energy reserves. In addition, we show that the combination of 4μ8C and DOX alters the inflammatory and fibrotic microenvironment. The long-term impact of our study could open the possibility of ER-stress inhibitors as adjuvant therapy for HCC-patients, as they could enhance the efficacy of DOX, for instance during TACE-treatment. This will be investigated in different dose-ratios in different preclinical HCC-models to be able to translate an efficient fixed dose combination to patients in the near future.

## Authors contributions

Conceptualization, F.H., M.K, H.L. and M.H.; resources F.H. and H.L.; writing—original draft preparation, M.K., D.B. and F.H. writing—review and editing, M.K. D.B. C.L.M, J.K., A.L.C., P.B, H.L., M.H. and F.H. Experimentation and procedures: M.K., D.B., C.L.M, J.K., A.L.C., S.S.N., data visualization, F.H.; supervision, F.H., M.K., M.H., P.B., F.R, C.E.B. and H.L.; project administration, F.H.; funding acquisition, F.H. and H.L. All authors have read and agreed to the published version of the manuscript.

## Declaration of competing interest

The authors declare that they have no known competing financial interests or personal relationships that could have appeared to influence the work reported in this paper.

## Figures and Tables

**Supplementary material 2:**
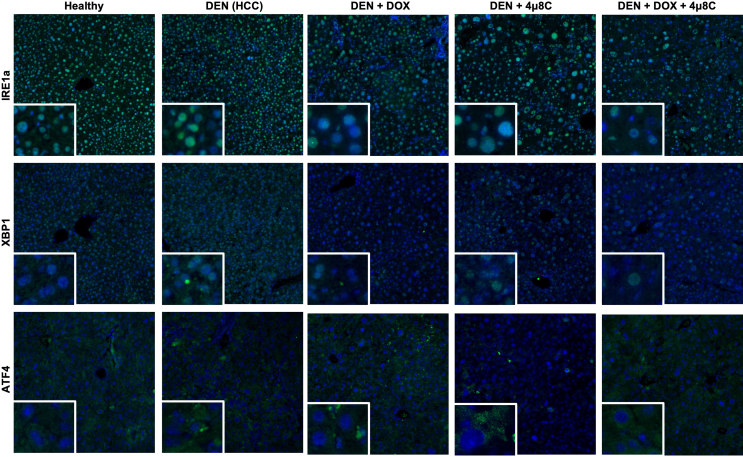
Representative images of fluorescent immunohistochemical stained liver slides, using antibodies against IRE1α, XBP1 and ATF4.

**Supplementary material 4:**
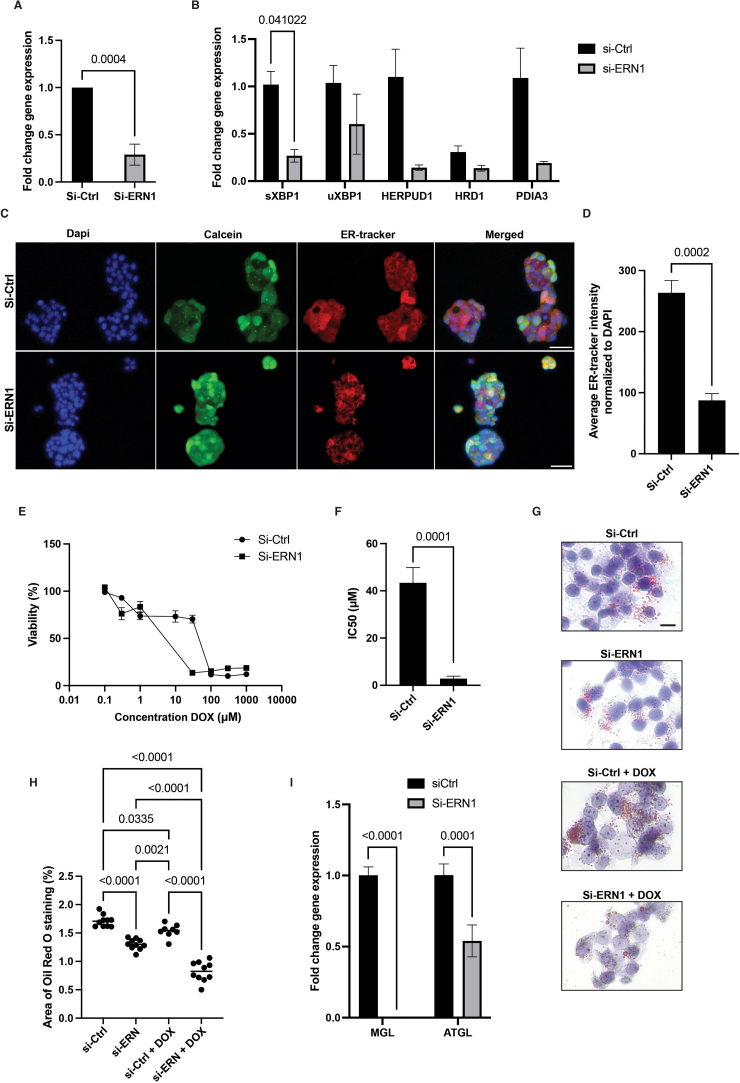
Silencing of ERN1 phenocopies the effect of pharmacological inhibition with 4μ8C. (A) mRNA-expression of ERN1. (B) mRNA expression of downstream targets of ERN1, namely sXBP1, uXBP1, HERPUD1, HRD1 and PDIA3. (C) Representative images of transfected and mock-transfected HepG2-cells stained with ER-stracker, Calcein and Dapi. (E) Cell viability of si-Ctrl and si-ERN1 HepG2-cells after treatment with various concentrations of DOX and (F) the resulting IC50 values were determined. (G) Representative images of Oil-Red-O staining. (H) Quantification of Oil-Red-O staining. (I) mRNA-expression of MGL and ATGL. N = 3–8 biological replicates.

**Supplementary material 5:**
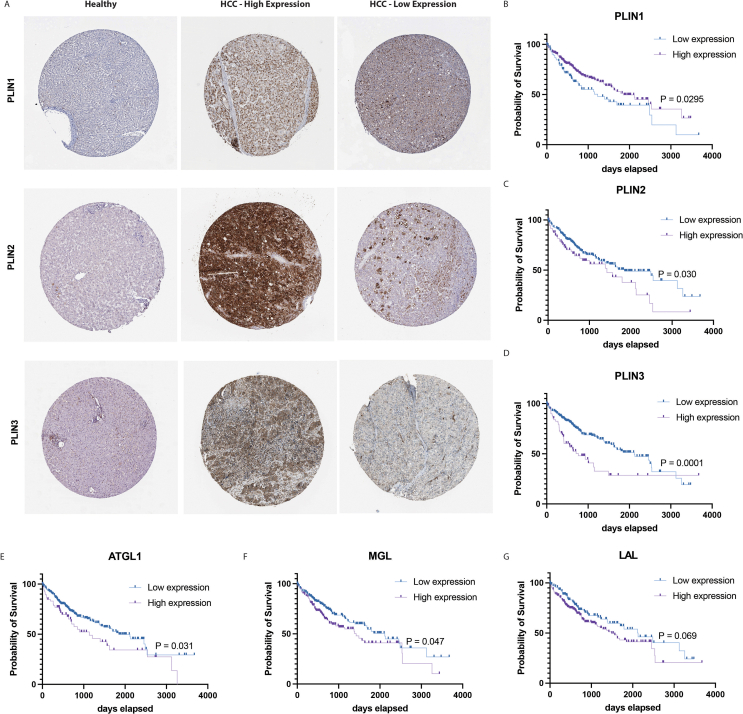
Expression levels of lipid accumulation markers and lipases correlate with poor prognosis in patients with HCC. (A) Immunohistochemical staining of PLIN1, PLIN2 and PLIN3 in HCC tissues. All images are obtained from the Human Protein Atlas database. Overall survival analysis of PLIN1 (B), PLIN2 (C), PLIN3 (D), ATGL1 (E), MGL (F) and ATGL (G). The association between the expression levels of these genes and overall survival of the HCC patients was analyzed by using the Kaplan–Meier plotter. Images and datasets are available from v23.proteinatlas.org.

## Data Availability

I have attached the raw data as supplementary material

## Appendix A Supplementary data

Supplementary material 1 Primer list. Supplementary material 1

Supplementary material 3 Raw data lipidomics. Supplementary material 3

## References

[1] Lohitesh K., Chowdhury R., Mukherjee S. (2018). Resistance a major hindrance to chemotherapy in hepatocellular carcinoma: an insight. Cancer Cell Int.

[2] Ebeling Barbier C., Heindryckx F., Lennernas H. (2021). Limitations and possibilities of transarterial chemotherapeutic treatment of hepatocellular carcinoma. Int J Mol Sci.

[3] Pavlović N., Heindryckx F. (2021). Exploring the role of endoplasmic reticulum stress in hepatocellular carcinoma through mining of the Human Protein Atlas. Biology.

[4] Ron D., Walter P. (2007). Signal integration in the endoplasmic reticulum unfolded protein response. Nat Rev Mol Cell Biol.

[5] Kaufman R.J. (2002). Orchestrating the unfolded protein response in health and disease. J Clin Invest.

[6] Wei C., Yang X., Liu N., Geng J., Tai Y., Sun Z. (2019). Tumor microenvironment regulation by the endoplasmic reticulum stress transmission mediator Golgi protein 73 in mice. Hepatology.

[7] Reibe S., Febbraio M.A. (2019). Relieving ER stress to target NASH-driven hepatocellular carcinoma. Nat Rev Endocrinol.

[8] Al-Rawashdeh F.Y., Scriven P., Cameron I.C., Vergani P.V., Wyld L. (2010). Unfolded protein response activation contributes to chemoresistance in hepatocellular carcinoma. Eur J Gastroenterol Hepatol.

[9] Shuda M., Kondoh N., Imazeki N., Tanaka K., Okada T., Mori K. (2003). Activation of the ATF6, XBP1 and grp78 genes in human hepatocellular carcinoma: a possible involvement of the ER stress pathway in hepatocarcinogenesis. J Hepatol.

[10] Bian X., Liu R., Meng Y., Xing D., Xu D., Lu Z. (2021). Lipid metabolism and cancer. J Exp Med.

[11] Hanahan D. (2022). Hallmarks of cancer: new dimensions. Cancer Discov.

[12] Germain N., Dhayer M., Boileau M., Fovez Q., Kluza J., Marchetti P. (2020). Lipid metabolism and resistance to anticancer treatment. Biology (Basel).

[13] Moncan M., Mnich K., Blomme A., Almanza A., Samali A., Gorman A.M. (2021). Regulation of lipid metabolism by the unfolded protein response. J Cell Mol Med.

[14] So J.S., Hur K.Y., Tarrio M., Ruda V., Frank-Kamenetsky M., Fitzgerald K. (2012). Silencing of lipid metabolism genes through IRE1alpha-mediated mRNA decay lowers plasma lipids in mice. Cell Metab.

[15] Heindryckx F., Mertens K., Charette N., Vandeghinste B., Casteleyn C., Van Steenkiste C. (2010). Kinetics of angiogenic changes in a new mouse model for hepatocellular carcinoma. Mol Cancer.

[16] Heindryckx F., Bogaerts E., Coulon S.H., Devlies H., Geerts A.M., Libbrecht L. (2012). Inhibition of the placental growth factor decreases burden of cholangiocarcinoma and hepatocellular carcinoma in a transgenic mouse model. Eur J Gastroenterol Hepatol.

[17] Nuciforo S., Fofana I., Matter M.S., Blumer T., Calabrese D., Boldanova T. (2018). Organoid models of human liver cancers derived from tumor needle biopsies. Cell Rep.

[18] Cui A., Hu Z., Han Y., Yang Y., Li Y. (2017). Optimized analysis of in vivo and in vitro hepatic steatosis. J Vis Exp.

[19] Balgoma D., Zelleroth S., Gronbladh A., Hallberg M., Pettersson C., Hedeland M. (2020). Anabolic androgenic steroids exert a selective remodeling of the plasma lipidome that mirrors the decrease of the de novo lipogenesis in the liver. Metabolomics.

[20] Balgoma D., Kullenberg F., Calitz C., Kopsida M., Heindryckx F., Lennernas H. (2021). Anthracyclins increase PUFAs: potential implications in ER stress and cell death. Cells.

[21] Karlsson M., Zhang C., Mear L., Zhong W., Digre A., Katona B. (2021). A single-cell type transcriptomics map of human tissues. Sci Adv.

[22] Heindryckx F., Coulon S., Terrie E., Casteleyn C., Stassen J.M., Geerts A. (2013). The placental growth factor as a target against hepatocellular carcinoma in a diethylnitrosamine-induced mouse model. J Hepatol.

[23] Andersen M.C.E., Johansen M.W., Nissen T., Nexoe A.B., Madsen G.I., Sorensen G.L. (2021). FIBCD1 ameliorates weight loss in chemotherapy-induced murine mucositis. Support Care Cancer.

[24] Atupelage C., Nagahashi H., Kimura F., Yamaguchi M., Tokiya A., Hashiguchi A. (2014). Computational hepatocellular carcinoma tumor grading based on cell nuclei classification. J Med Imaging (Bellingham).

[25] Heindryckx F., Binet F., Ponticos M., Rombouts K., Lau J., Kreuger J. (2016). Endoplasmic reticulum stress enhances fibrosis through IRE1alpha-mediated degradation of miR-150 and XBP-1 splicing. EMBO Mol Med.

[26] Pavlovic N., Kopsida M., Gerwins P., Heindryckx F. (2020). Inhibiting P2Y12 in macrophages induces endoplasmic reticulum stress and promotes an anti-tumoral phenotype. Int J Mol Sci.

[27] Abdelaziz A.O., Abdelhalim H., Elsharkawy A., Shousha H.I., Abdelmaksoud A.H., Soliman Z.A. (2019). Liver stiffness measurement changes following hepatocellular carcinoma treatment with percutaneous microwave ablation or transarterial chemoembolization: a cohort study. Eur J Gastroenterol Hepatol.

[28] O'Rourke J.M., Sagar V.M., Shah T., Shetty S. (2018). Carcinogenesis on the background of liver fibrosis: implications for the management of hepatocellular cancer. World J Gastroenterol.

[29] Uemura A., Oku M., Mori K., Yoshida H. (2009). Unconventional splicing of XBP1 mRNA occurs in the cytoplasm during the mammalian unfolded protein response. J Cell Sci.

[30] Jiang D., Lynch C., Medeiros B.C., Liedtke M., Bam R., Tam A.B. (2016). Identification of doxorubicin as an inhibitor of the IRE1alpha-XBP1 axis of the unfolded protein response. Sci Rep.

[31] Kim S.J., Park K.M., Kim N., Yeom Y.I. (2006). Doxorubicin prevents endoplasmic reticulum stress-induced apoptosis. Biochem Biophys Res Commun.

[32] Finley L.W.S. (2023). What is cancer metabolism?. Cell.

[33] Shaffer A.L., Shapiro-Shelef M., Iwakoshi N.N., Lee A.H., Qian S.B., Zhao H. (2004). XBP1, downstream of Blimp-1, expands the secretory apparatus and other organelles, and increases protein synthesis in plasma cell differentiation. Immunity.

[34] Chen Y., Mi Y., Zhang X., Ma Q., Song Y., Zhang L. (2019). Dihydroartemisinin-induced unfolded protein response feedback attenuates ferroptosis via PERK/ATF4/HSPA5 pathway in glioma cells. J Exp Clin Cancer Res.

[35] Ohyashiki T., Kadoya A., Kushida K. (2002). The role of Fe3+ on Fe2+-dependent lipid peroxidation in phospholipid liposomes. Chem Pharm Bull (Tokyo).

[36] Yoshida G.J. (2020). The interplay between apoptosis and ferroptosis mediated by ER stress. Apoptosis.

[37] Han J., Kaufman R.J. (2016). The role of ER stress in lipid metabolism and lipotoxicity. J Lipid Res.

[38] Luna-Marco C., Ubink A., Kopsida M., Heindryckx F. (2023). Endoplasmic reticulum stress and metabolism in hepatocellular carcinoma. Am J Pathol.

[39] De Matteis S., Ragusa A., Marisi G., De Domenico S., Casadei Gardini A., Bonafe M. (2018). Aberrant metabolism in hepatocellular carcinoma provides diagnostic and therapeutic opportunities. Oxid Med Cell Longev.

[40] Zhang K., Wang S., Malhotra J., Hassler J.R., Back S.H., Wang G. (2011). The unfolded protein response transducer IRE1alpha prevents ER stress-induced hepatic steatosis. EMBO J.

[41] Zechner R., Madeo F., Kratky D. (2017). Cytosolic lipolysis and lipophagy: two sides of the same coin. Nat Rev Mol Cell Biol.

[42] Kinoshita M., Uchida T., Sato A., Nakashima M., Nakashima H., Shono S. (2010). Characterization of two F4/80-positive Kupffer cell subsets by their function and phenotype in mice. J Hepatol.

[43] Ren C.X., Leng R.X., Fan Y.G., Pan H.F., Li B.Z., Wu C.H. (2017). Intratumoral and peritumoral expression of CD68 and CD206 in hepatocellular carcinoma and their prognostic value. Oncol Rep.

[44] Yang F., Wei Y., Han D., Li Y., Shi S., Jiao D. (2020). Interaction with CD68 and regulation of GAS6 expression by endosialin in fibroblasts drives recruitment and polarization of macrophages in hepatocellular carcinoma. Cancer Res.

[45] Silva-Cardoso S.C., Tao W., Angiolilli C., Lopes A.P., Bekker C.P.J., Devaprasad A. (2020). CXCL4 links inflammation and fibrosis by reprogramming monocyte-derived dendritic cells in vitro. Front Immunol.

