# Supplementary material for: Inhibiting the endoplasmic reticulum stress response enhances the effect of doxorubicin by altering the lipid metabolism of liver cancer cells
Source: Mol Metab. 2023 Nov 27;79:101846. doi: 10.1016/j.molmet.2023.101846 (PMC10755054; doi:10.1016/j.molmet.2023.101846)
Supplement: Supplementary material 1 — Primer list. [file mmc1.docx]

Supplementary Material 1: primer list

| Human | 18S rRNA | F: 5’ AGTCCCTGCCCTTTGTACACA 3’  R: 5’ GATCCGAGGGCCTCACTAAAC 3’ |
| --- | --- | --- |
| Human | ATGL | F: 5’ ACCATCCGGTGTGAGGGC 3’  R: 5’ TCCCTGGTAACCATCACTCG 3’ |
| Human | MGL | F: 5’ GAATGCAAACGCCAGCACATA 3’  R: 5’ ACAAAGATGAGGGCCTTGGGTG 3’ |
| Human | LAL | F: 5’ CCAACCCCAAGAACCGCATT 3’  R: 5’ CCTTTCACCTTTTACTTGCCCTAAA 3’ |
| Human | EDEM1 | F: 5’ ACGAGCAGTGAAAGCCCTTTGG3’  R: 5’ CCACTCTGCTTTCCAACCCAGT3’ |
| Human | HERPUD1 | F: 5’ CCAATGTCTCAGGGACTTGCTTC3’  R: 5’ CGATTAGAACCAGCAGGCTCCT3’ |
| Human | HRD1 | F: 5’CCAACATCTCCTGGCTCTTTCAC3’  R: 5’GTCAGGATGCTGTGATAGGCGT3’ |
| Human | PDIA3 | F: 5’ GTCAGCCACTTGAAGAAGCAGG3’  R: 5’ TAGGAACTCGGAGTGAGCCTCA3’ |
| Mouse | GAPDH | F: 5’AATGAAGGGGTCGTTGATG3’ R: 5’GGTGAAGGTCGGTGTGAAC3’ |
| Mouse | ATGL | F: 5´TCGTGTTTCAGACGGAGAGAA 3´  R: 5´ CAGACATTGGCCTGGATGAG 3´ |
| Mouse | MGL | F: 5´CCAGGCGAACTCCACAGAAT 3´  R: 5´GAGAGGCAGGTCACGGAAAG 3´ |
| Mouse | LAL | F: 5´AGCTTGCCCGAATTGTATGTG 3´  R: 5´TCTGTTGTGAACCATGTGACTTC 3´ |
| Mouse | MGL-CoAR | F: 5´AGCTTGCCCGAATTGTATGTG 3´  R: 5´TCTGTTGTGAACCATGTGACTTC 3´ |
| Mouse | PPAR | F: 5´CTGCCTTCCCTGTGAACTGA 3´  R: 5´ACAGAGCGCTAAGCTGTGAT 3´ |
| Mouse | PCNA | F: 5´ AGGCACTCAAGGACCTCATCA 3´ R: 5´GAGTCCATGCTCTGCAGGTTT 3´ |
| Mouse | XBP1 s | F 5´CTGAGTCCGAATCAGGTGCAG 3´  R 5´GTCCATGGGAAGATGTTCTGG 3´ |
| Mouse | XBP1 u | F: 5´ CAGCACTCAGACTATGTGCA 3´  R: 5´ GTCCATGGGAAGATGTTCTGG 3´ |
| Mouse | CHOP | F: 5´CATCACCACACCTGAAAGCA 3´  F: 5´ TCAGCTGCCATCTCTGCA 3´ |
| Mouse | PERK | F CCTAGATTCTGAGTCGGGAGG  R CACAGATGTCCAGGCCACAA |
| Mouse | ATF6 | F 5´CCATCCGAGTTGTGAGGGAG 3´  R 5´TCGGGTACATTCGCAGATCC 3´ |
| Mouse | aSMA | F: 5’- CTGACAGAGGCACCACTGAA-3’  R: 5’- CATCTCCAGAGTCCAGCACA-3’ |
| Mouse | CXCL4 | F: 5´TCAGTGGCTGACCTCCTCTT 3´  R: 5´CTTGGCCTTTGACTGTTGGT 3´ |
| Mouse | CD68 | F: 5´ACCGCCATGTAGTCCAGGTA 3´  R: 5´ATCCCCACCTGTCTCTCTCA 3´ |
| Mouse | ATF4 | F 5´CTTGATGTCCCCCTTCGACC3´  R 5´GGGAGGCAGAAAGCCATTAGA 3´ |
| Mouse | EDEM1 | F: 5’GCTGCGTATCAGAGCATCCAGA3’  R: 5’CAGCGAGTCAATCCAGGTGTTC3’ |
| Mouse | HERPUD1 | F: 5’CCTCCAAAATGCCAGAAACCAGC3’  R: 5’GCCGTAAACCATCACTTGAGGAG3’ |
| Mouse | HRD1 | F: 5’CCAACATCTCCTGGCTCTTCCA3’  R: 5’CAGGATGCTGTGATAAGCGTGG3’ |
| Mouse | DNAJB9 | F: 5’AGCCATGAAGTACCACCCTGAC3’  R: 5’CGACTATTGGCATCCGAGAGTG3’ |
| Mouse | DNAJB11 | F: 5’TGTGACCGTCTCACTGGTTGAG3’  R: 5’CCCTTTCTTCCACAGCTTGGCT3’ |
| Mouse | PDIA3 | F: 5’ TGTGACCGTCTCACTGGTTGAG3’  R: 5’ CCCTTTCTTCCACAGCTTGGCT3’ |
